# Estimation of Heterogeneous Restricted Mean Survival Time Using Random Forest

**DOI:** 10.3389/fgene.2020.587378

**Published:** 2021-01-07

**Authors:** Mingyang Liu, Hongzhe Li

**Affiliations:** Department of Biostatistics, Epidemiology, and Informatics, Perelman School of Medicine, University of Pennsylvania, Philadelphia, PA, United States

**Keywords:** estimating equation, high dimensional data, non-parametric survival estimation, regression forest, inference

## Abstract

Estimation and prediction of heterogeneous restricted mean survival time (hRMST) is of great clinical importance, which can provide an easily interpretable and clinically meaningful summary of the survival function in the presence of censoring and individual covariates. The existing methods for the modeling of hRMST rely on proportional hazards or other parametric assumptions on the survival distribution. In this paper, we propose a random forest based estimation of hRMST for right-censored survival data with covariates and prove a central limit theorem for the resulting estimator. In addition, we present a computationally efficient construction for the confidence interval of hRMST. Our simulations show that the resulting confidence intervals have the correct coverage probability of the hRMST, and the random forest based estimate of hRMST has smaller prediction errors than the parametric models when the models are mis-specified. We apply the method to the ovarian cancer data set from The Cancer Genome Atlas (TCGA) project to predict hRMST and show an improved prediction performance over the existing methods. A software implementation, srf using R and C++, is available at https://github.com/lmy1019/SRF.

## 1. Introduction

In epidemiological and biomedical studies, time to an event or survival time *T* is often the primary outcome of interest. Important quantities related to survival time include hazard rate (HR), *t*-year survival probability, and the mean survival time. Among these, HR is one of the most commonly used quantity due to its strong connection to the proportional hazards regression model or Cox model. Cox model is a very popular regression model for censored survival data due to its computational feasibility and theoretical properties (Cox, 1972, 1975; Andersen and Gill, 1982; Gill and Gill, 1984; Huang et al., 2013; Fang et al., 2017). However, when there is a departure from the proportional hazards assumption, the connection between HR and survival function is lost and it is difficult to interpret HR (Wang and Schaubel, 2018). The *t*-year survival probability is the probability of survival time greater than a pre-specified time *t*. It is not suitable for summarizing the global profile of *T* over the duration of a study (Tian et al., 2014). In contrast, mean survival time is an alternative quantity since it takes the whole distribution of *T* into account. However, the mean of *T* may not always be estimable in the presence of censoring. For example, let *C* denotes the censoring time, and $C_{\max}=\inf_{c}\left\{P\left(C\leq c\right)=1\right\}$ be the upper limit of the censoring distribution,

$$\begin{array}{l} E_{T}\left[T\right]=E_{T}\left[T|T\leq C_{\max}\right]P\left(T\leq C_{\max}\right)\\ \;+\;E_{T}\left[T|T>C_{\max}\right]P\left(T>C_{\max}\right) \end{array}$$

If the survival time *T* satisfies *P*(*T* > *C*_max_) > 0, then we cannot estimate *E*_*T*_[*T*], since we never observe any event after *C*_max_.

The restricted mean survival time (RMST) (Royston and Parmar, 2013) summarizes the survival process and provides an attractive alternative to the proportional hazards regression model (Tian et al., 2014). The restricted survival time of *T* up to a fixed point *L* is defined as *T* ∧ *L*, and the restricted mean survival time is defined as the expectation of the restricted survival time. Denote μ^*L*^(*x*) = *E*[*T* ∧ *L*|*X* = *x*] be the heterogeneous RMST with covariates *X* = *x*. It can be written as the area under the survival curve on [0, *L*].

(1) $$\begin{array}{l} \begin{array}{l} \mu^{L}\left(x\right)=\int_{0}^{\infty }\left(\int_{0}^{\infty }1_{u<t}1_{u<L}du\right)\;f_{T}\left(t|X=x\right)dt\\ \;=\int_{0}^{L}S\left(u|X=x\right)du. \end{array} \end{array}$$

If *L* is chosen to be less than *C*_max_, hRMST is estimable since *P*(*T* ∧ *L* > *C*_max_) = 0. RMST also plays a role in the context of inverse probability censoring weighting (IPCW). A key assumption for applying IPCW is *P*(*T* < *C*_max_) = 1, making 1/(1 − *G*(*T*)) well-defined, where *G*(*T*) = *P*(*C* ≤ *T*|*T*). If we set *L* properly such that *P*(*T* ∧ *L* < *C*_max_) = 1, then *G*(*T* ∧ *C* ∧ *L*|*X*) < 1 and the IPCW is well-defined under the restricted survival time context.

**Figure 1:**
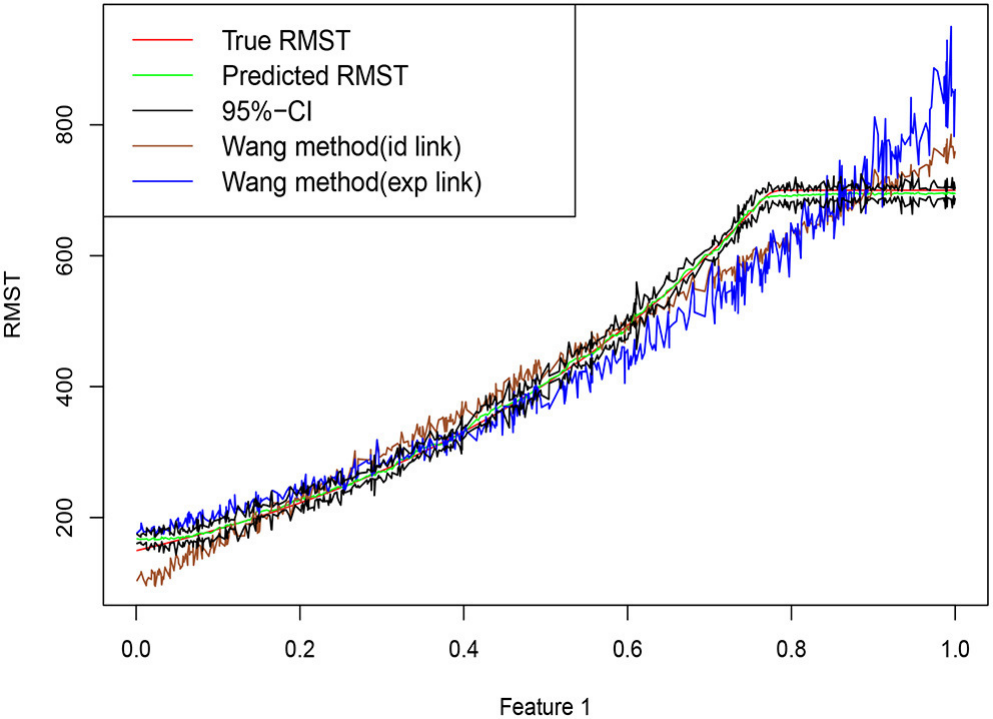
Training data are simulated from Equation (2), with *n* = 600 training points, dimension *p* = 20 and errors ϵ ~ *N*(0, 10^2^). Random forests are trained based using R package grf. Truth is shown as red curve, with green curve corresponding to the random forest predictions, and upper and lower bounds of the point-wise confidence intervals connected in the black lines. Brown curve and blue curve are based on the approaches of Wang and Schaubel (2018) with Identity and Exp link functions.

There are two main approaches for hRMST regression. One approach is to estimate hRMST indirectly through hazard regression (Zucker, 1998; Chen and Tsiatis, 2001; Zhang and Schaubel, 2011). This approach starts by estimating the regression parameters and the baseline hazard from a Cox model, calculating the cumulative baseline hazard, transforming it to obtain the survival function and, finally, obtaining the hRMST through Equation (1). Such an indirect hRMST estimation is inconvenient and computationally cumbersome for obtaining a point estimate and its corresponding asymptotic standard error. An alternative approach is to model hRMST with the baseline covariates *X* directly via some parametric assumptions, eg. $g\left[\mu^{L}\left(X_{i}\right)\right]=\beta_{0}^{\prime }X_{i},$ where *g* is a strictly monotone link function with a continuous derivative within an open neighborhood (Tian et al., 2014; Wang and Schaubel, 2018). A major weakness of this approach, however, is their inability to choose a proper link function, which may lead to the model misspecification. As an example, we simulate *x*_1_, …, *x*_*n*_ independently from the uniform distribution on [0, 1]^20^ with a survival time model

(2) $$\begin{array}{l} T=\exp\left(2X_{1}+5\right)+1+\epsilon ,\;\epsilon \sim N\left(0,10^{2}\right), \end{array}$$

where we assume that the censoring time *C* and the restricted time *L* satisfy *P*(*C* ≤ *T* ∧ *L*) = 33% and *P*(*L* ≤ *T* ∧ *C*) = 11%. Our goal is to estimate μ^*L*^(*x*). Figure 1 shows a set of predictions on an artificially generated data set from Equation (2). Compared with other methods, the random forest is able to estimate the target function closely, especially when μ^*L*^(*x*) approaches *L*.

For the continuous outcomes without censoring, random forest (Breiman, 2001, 2004) is a popular method of non-parametric regression that has shown effectiveness in many applications (Svetnik et al., 2003; Díaz-Uriarte and Alvarez de Andrés, 2006; Cutler et al., 2007). It is invariant under scaling and various other transformations of feature values, robust to inclusion of irrelevant features (Hastie et al., 2001), and versatile enough to be applied to large-scale problems (Biau and Scornet, 2016). Besides strong empirical results, theoretical results such as consistency (Meinshausen, 2006; Biau et al., 2008; Biau, 2012; Denil et al., 2014) and asymptotic normality (Wager and Athey, 2015; Mentch and Hooker, 2016; Athey et al., 2018; Friedberg et al., 2018) have also been obtained for regression models without censoring. Extending random forest to censored survival data has been proposed in several recent papers (Ishwaran et al., 2008; Steingrimsson et al., 2019), focusing on implementations and algorithms. However, there has been little theoretical work in statistical inference of such random survival forest. Ishwaran and Kogalur (2011) proved the consistency of the random survival forest by showing that the forest ensemble survival function converges uniformly to the true population survival function.

Instead of focusing on predicting the survival function or the survival probability as the algorithms implemented by Ishwaran et al. (2008) and Steingrimsson et al. (2019), we develop in this paper a random forest framework to model the hRMST directly given the baseline covariates in the presence of possibly covariate-dependent censoring. This approach provides a non-parametric estimation of hRMST adjusting for covariates. Due to the complex relationship between the survival time and the covariates, it is desirable to have more flexible methods to estimate the hRMST than the approaches that a certain link function has to be assumed. Our construction of random forest is based on the estimated IPCW. We show that the resulting survival random forest estimates of hRMST has the asymptotic normality property that can be used to obtain the point-wise confidence interval with theoretical guarantees. To the best of our knowledge, it is the first asymptotic normality result for the predictions in the context of censored survival data using random forest.

The remainder of the paper is organized as follows. In section 2, we describe the proposed random forest estimator. Asymptotic properties are given in section 3. In section 4, we conduct simulation studies to evaluate the accuracy of the proposed method in the finite sample settings. In section 5, we apply our method to an ovarian cancer data set of The Cancer Genome Atlas (TCGA) project (http://cancergenome.nih.gov/abouttcga) to evaluate the predictions of the hRMST for ovarian cancer patients using their acylcarnitine measurements and clinical variables. We conclude this chapter with a brief discussion in section 6.

## 2. Random Forest for Estimating the hRMST

We begin with some notation. Let *X*_*i*_ be the baseline covariates for subject *i* from a cohort of sample size *n* and *T*_*i*_ be the survival time for subject *i*. Let *C*_*i*_ be the censoring time, which is independent of *T*_*i*_ conditional on the baseline covariates *X*_*i*_. The observation time for subject *i* is *Z*_*i*_ = *T*_*i*_ ∧ *C*_*i*_, where *a* ∧ *b* = min{*a, b*}. The indicator for censoring is denoted by δ_*i*_ = 1_{_*T*__*i*_ ≤ *C*_*i*_}_. Our observed *i*.*i*.*d*. data are given as {(*X*_*i*_, *Z*_*i*_, δ_*i*_):*i* = 1, …, *n*}.

Let *L* be a pre-specified time point of interest, before the maximum follow-up time τ = max{*Z*_*i*_ : *i* = 1, …, *n*}. As in Wang and Schaubel (2018), *L* is normally chosen as a time point of clinical relevance or, at least, of particular interest to the investigators, respecting the bound at the maximum follow-up time. Denote the restricted observation time as $Z_{i}^{L}=Z_{i}\wedge L$ and its corresponding indicator $\delta_{i}^{L}=1_{\left\{T_{i}\wedge L\leq C_{i}\right\}}$. Our goal is to estimate covariate-adjusted RMST or hRMST μ^*L*^(*x*) = *E*(*Z*^*L*^|*X* = *x*) and to construct its confidence interval.

### 2.1. Forest-Based Local Estimating Equation for hRMST

Given the observed data ${\left\{\left(X_{i},\delta_{i},Z_{i}\right)\right\}}_{i=1}^{n}$, and a restriction threshold *L*, we first present a random forest method to estimate μ^*L*^(*x*). The idea of the approach is to solve a weighted estimating equation for μ^*L*^(*x*), where the estimating equation functions of the observations whose covariates closer to *x* will have larger weights. Specifically, let $w_{i}=\delta_{i}^{L}/\left(1-G\left(Z_{i}^{L}|X_{i}\right)\right)$ be the IPCW of the *i*th data point under the true censoring distribution *G*(·|*X*_*i*_). The (infeasible) estimating equation function $w_{i}\left(Z_{i}^{L}-\mu^{L}\left(x\right)\right)$ of *X*_*i*_ = *x* satisfies $E\left[w_{i}\left(Z_{i}^{L}-\mu^{L}\left(x\right)\right)|X_{i}=x\right]=E\left[T_{i}\wedge L|X_{i}=x\right]-\mu^{L}\left(x\right)=0$. If the local weights ${\left\{\alpha_{i}\left(x\right)\right\}}_{i=1}^{n}$ are also known, the solution to the empirical estimating equation for μ^*L*^(*x*)

(3) $$\begin{array}{l} \begin{array}{c} \overset{n}{\underset{i=1}{\sum }}\alpha_{i}\left(x\right)w_{i}\left(Z_{i}^{L}-\mu \right)=0 \end{array} \end{array}$$

is given as

$$\frac{\sum_{i=1}^{n}\alpha_{i}\left(x\right)w_{i}Z_{i}^{L}}{\sum_{i=1}^{n}\alpha_{i}\left(x\right)w_{i}},$$

which provides a good candidate of estimator for μ^*L*^(*x*). However we do not know the censoring distribution *G* and the local weights ${\left\{\alpha_{i}\left(x\right)\right\}}_{i=1}^{n}$, which need to be estimated from the data. We assume censoring distribution *G* follows a Cox model, a natural choice for modeling censoring times in the context of IPCW. Let

$${\hat{w}}_{i}=\frac{\delta_{i}^{L}}{1-\hat{G}\left(Z_{i}^{L}|X_{i}\right)}$$

be the estimated IPCW for *i*th observation with Ĝ(·|*X*_*i*_) derived from the data through Cox model. We define the estimating equation function for *i*th observation with its corresponding estimated IPCW as

$$\psi_{\mu^{L}\left(x\right)}\left(X_{i},Z_{i}^{L},\delta_{i}^{L}\right)={\hat{w}}_{i}\left(Z_{i}^{L}-\mu_{i}^{L}\left(x\right)\right).$$

Our approach to derive the local weights ${\left\{\alpha_{i}\left(x\right)\right\}}_{i=1}^{n}$ is through the random forest, which is an ensemble of survival trees constructed by Algorithm 1.

**Algorithm 1:**
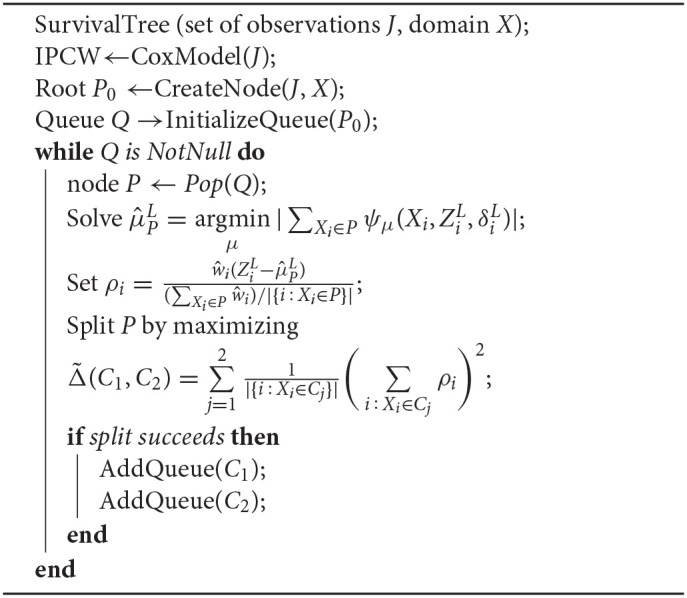
Survival tree

It can be shown that ρ_*i*_ is the influence function of the *i*th observation for ${\hat{\mu }}_{P}^{L}$. Let *F*_*n*_ be the empirical distribution of the observations in node *P*, and let *F*_*n,i*_ = (1 − ϵ)*F*_*n*_ + ϵ ν_*i*_, with ν_*i*_ be the Dirac delta function at *i*th observation. Set ${\hat{\mu }}_{P,i}^{L}={\hat{\mu }}_{P}^{L}+\Delta_{i}$, where ${\hat{\mu }}_{P,i}^{L}=\begin{array}{c} argmin\\ \mu \end{array}|\int \psi_{\mu }\left(X,Z^{L},\delta^{L}\right)dF_{n,i}|$. By Taylor expansion,

$$\begin{array}{l} 0=\int \psi_{{\hat{\mu }}_{P,i}^{L}}\left(X,Z^{L},\delta^{L}\right)dF_{n,i}\\ \;=\int \left[\psi_{{\hat{\mu }}_{P}^{L}}\left(X,Z^{L},\delta^{L}\right)+\psi_{\mu^{*}}^{\prime }\left(X,Z^{L},\delta^{L}\right)\Delta_{i}\right]dF_{n,i}, \end{array}$$

where μ^*^ is a value between ${\hat{\mu }}_{P}^{L}$ and ${\hat{\mu }}_{P,i}^{L}$. The above equation implies

$$\Delta_{i}=-\frac{\epsilon \psi_{{\hat{\mu }}_{P}^{L}}\left(X_{i},Z_{i}^{L},\delta_{i}^{L}\right)}{\int \psi_{\mu^{*}}^{\prime }\left(X,Z^{L},\delta^{L}\right)dF_{n,i}},$$

and therefore the influence function of *i*th observation for ${\hat{\mu }}_{P}^{L}$ is

$$\underset{\epsilon \to 0}{\lim}\Delta_{i}/\epsilon =-\frac{\psi_{{\hat{\mu }}_{P}^{L}}\left(X_{i},Z_{i}^{L},\delta_{i}^{L}\right)}{\int \psi_{{\hat{\mu }}_{P}^{L}}^{\prime }\left(X,Z^{L},\delta^{L}\right)dF_{n}}=\frac{{\hat{w}}_{i}\left(Z_{i}^{L}-{\hat{\mu }}_{P}^{L}\right)}{\sum_{i\in P}\frac{{\hat{w}}_{i}}{\left|\left\{i:X_{i}\in P\right\}\right|}}=\rho_{i}.$$

Athey et al. (2018) shows that maximizing the splitting criterion $\tilde{\Delta }\left(C_{1},C_{2}\right)$ is approximately equivalent to minimizing the weighted mean squared error $err\left(C_{1},C_{2}\right)=\underset{i=1,2}{\sum }P\left(X\in C_{i}|X\in P\right)E\left[{\left({\hat{\mu }}_{C_{i}}^{L}-\mu^{L}\left(X\right)\right)}^{2}|X\in C_{i}\right]$.

In order to achieve consistency and asymptotic normality, we split the tree and make predictions in an honest way as introduced in Wager and Athey (2015). Specifically, each tree in an honest forest is grown using two non-overlapping subsamples of the training data. For the *b*th tree, given *I*_*b*_ and *J*_*b*_, we first choose the tree structure *T*_*b*_ using only the data in *J*_*b*_, and write $x{↔}_{b}x^{\prime }$ as the boolean indicator for whether the points *x* and *x*′ fall into the same leaf of *T*_*b*_. In a second step, we define the set of neighbors of *x* as *L*_*b*_(*x*) = {*i* ∈ *I*_*b*_ : *x* ↔ _*b*_*x*_*i*_}. The weights of point *x* from a survival forest with *B* trees can be written as

$$\alpha_{i}\left(x\right)=\frac{1}{B}\overset{B}{\underset{b=1}{\sum }}\frac{1_{\left\{X_{i}\in L_{b}\left(x\right)\right\}}}{\left|L_{b}\left(x\right)\right|}.$$

The empirical locally weighted estimating equation for ${\hat{\mu }}^{L}\left(x\right)$ is then defined as

(4) $$\begin{array}{l} \overset{n}{\underset{i=1}{\sum }}\alpha_{i}\left(x\right)\psi_{\mu }\left(X_{i},Z_{i}^{L},\delta_{i}^{L}\right)=0, \end{array}$$

and the random forest estimator for the hRMST is the solution of Equation (4), which is

$${\hat{\mu }}^{L}\left(x\right)=\overset{n}{\underset{i=1}{\sum }}\frac{\alpha_{i}\left(x\right){\hat{w}}_{i}Z_{i}^{L}}{\sum_{i=1}^{n}\alpha_{i}\left(x\right){\hat{w}}_{i}}.$$

We emphasize the difference between the IPCW used in building the survival trees and IPCW used to derive ${\hat{\mu }}^{L}\left(x\right)$. The IPCW used in building survival trees is estimated only by the data points from *J*_*b*_ so that the resulting survival forest is honest. The IPCW used to derive ${\hat{\mu }}^{L}\left(x\right)$ is estimated from all data points.

## 3. Asymptotic Distribution of ${\hat{\mu }}^{L}\left(x\right)$

### 3.1. Asymptotic Normality

We derive a central limit theorem for survival forest estimate of hRMST. We first give three common assumptions that required for the most of the theoretical analysis of random forests.

**Assumption 1**. μ^*L*^(*x*) *is Lipschitz continuous w.r.t x*.

**Assumption 2**. *There exists a restricted time threshold L*, *such that P*(*C* > *t* ∧ *L*|*X* = *x*) ≥ ϵ_*L*_ > 0 *for any x, t*.

**Assumption 3**. *Var*(*T* ∧ *L*|*X* = *x*) > 0 *for any x*.

As mentioned in the previous section, we model the conditional survival function of censoring distribution *G* given baseline covariates. Because of its flexibility and popularity in practice, we adopt the proportional hazards model for hazard function of censoring distribution.

**Assumption 4**. *The hazard function of censoring distribution follows* $\lambda_{i}^{C}\left(t\right)=\lambda_{0}^{C}\left(t\right)\exp\left(X_{i}^{\prime }\beta_{C}\right)$

We make additional regularity assumptions that are widely used in analysis of estimates from the proportional hazards models. These assumptions are needed in order to quantify the difference between the estimated IPCW and true IPCW.

**Assumption 5**. ‖*X*‖_∞_ < *M*_*X*_ < ∞

**Assumption 6**. $\lambda_{0}^{C}\left(t\right)\leq \lambda_{0}^{C}<\infty$ *for all t*.

**Assumption 7**. $\Omega_{C}\left(\beta \right)=E\left[\int_{0}^{\tau }\frac{r^{\left(2\right)}\left(t,\beta \right)}{r^{\left(0\right)}\left(t,\beta \right)}-\bar{x}{\left(t,\beta \right)}^{⊗2}dN_{i}^{C}\left(t\right)\right]$ *is positive definite, where R*_*i*_(*t*) = 1(*Z*_*i*_ ≥ *t*), $r^{\left(k\right)}\left(t,\beta \right)=E\left[\exp\left(\beta^{\prime }X_{i}\right)R_{i}\left(t\right)X_{i}^{⊗k}\right]$, $\bar{x}\left(t,\beta \right)=\frac{r^{\left(1\right)}\left(t,\beta \right)}{r^{\left(0\right)}\left(t,\beta \right)},N_{i}^{C}\left(t\right)=1_{Z_{i}\leq t,\delta_{i}=0}$.

**Assumption 8**. *P*(*R*_*i*_(*t*) = 1|*X*_*i*_ = *x*) ≥ *r* > 0 *for some positive constant and for any t, x. This assumption implies that*

$$r^{\left(0\right)}\left(t,\beta \right)=E\left[\exp\left(\beta^{\prime }X_{i}\right)R_{i}\left(t\right)\right]=E\left[\exp\left(\beta^{\prime }X_{i}\right)E\left[R_{i}\left(t\right)|X_{i}\right]\right]\geq r>0.$$

Following Wager and Athey (2015) and Athey et al. (2018), we assume that all trees are symmetric, in that their output is invariant to permuting the indices of Estimation-Part in training examples (see Corollary 6 of Wager and Athey (2015) for more details about this symmetry). They also require balanced splits in the sense that every split puts at least a fraction ω of the observations in the parent node into each child, for some ω > 0. Finally, the trees are randomized in such a way that, at every split, the probability that the tree splits on the *j*th feature is bounded from below by some π > 0. The forest is honest and built via subsampling with subsample size s satisfying *s*/*n* → 0 and *s* → ∞.

Under the assumptions listed above, we have the following asymptotic distribution result for the random forest-based estimate of the hRMST.

**Theorem 1**. *Under Assumptions* 1, 2, 3, 4, 5, 6, 7, 8, *for each fixed test point x*, *there is a sequence* $\sigma_{n}^{2}\left(x\right)=Var\left({\hat{\mu }}^{L}\left(x\right)\right)\to 0$,

$$\frac{{\hat{\mu }}^{L}\left(x\right)-\mu^{L}\left(x\right)}{\sigma_{n}\left(x\right)}\to_{d}N\left(0,1\right)$$

if subsampling size

$$\beta_{\min}=1-(1+\frac{\pi^{-1}(\log\left(\omega^{-1}\right))}{\log({\left(1-\omega \right)}^{-1})}{)}^{-1},$$

*where ω > 0 is the low-bound fraction for observations in the parent node into each child, and π > 0 is the lower-bound of the probability that the tree splits on any features*.

We give a consistent estimate of $\sigma_{n}^{2}\left(x\right)$ based on half-sampling (Efron, 1980) and the method of Sexton and Laake (2009).

### 3.2. Estimation of the Variance

Following Athey et al. (2018), we use the random forest delta method to develop a variance estimate of the survival forest prediction ${\hat{\mu }}^{L}\left(x\right)$. Athey et al. (2018) provides a consistent estimate of $\sigma_{n}^{2}\left(x\right)$ using $s_{n}^{2}\left(x\right)$, where $s_{n}^{2}\left(x\right)=\left(V{\left(x\right)}^{-1}\right)H_{n}\left(x\right){\left(V{\left(x\right)}^{-1}\right)}^{\prime }$ with

$$\begin{array}{l} H_{n}\left(x\right)=Var\left[\overset{n}{\underset{i=1}{\sum }}\alpha_{i}\left(x\right)\psi_{\mu^{L}\left(x\right)}\left(X_{i},Z_{i}^{L},\delta_{i}^{L}\right)\right]\\ \;V\left(x\right)=\frac{\partial }{\partial \left(\mu^{L}\right)}E\left[\psi_{\mu^{L}}\left(X,Z^{L},\delta^{L}\right)|X=x\right]{|}_{\mu^{L}=\mu^{L}\left(x\right)} \end{array}$$

In our context, *V*(*x*) = − 1, then simply we have $s_{n}^{2}\left(x\right)=H_{n}\left(x\right)$.

A consistent estimator for *H*_*n*_(*x*) can be obtained using half-sampling estimator (Efron, 1980; Athey et al., 2018). Let ${\text{Ψ}}_{H}$ be the average of the empirical estimating equation functions averaged over the trees that only use the data from the half-sample H, denoted by $S_{H}$,

$${\text{Ψ}}_{H}\left(x\right)=\frac{1}{\left|S_{H}\right|}\underset{b\in S_{H}}{\sum }\frac{\sum_{i=1}^{n}1_{X_{i}\in L_{b}\left(x\right)}\psi_{{\hat{\mu }}^{L}\left(x\right)}\left(X_{i},Z_{i}^{L},\delta_{i}^{L}\right)}{\sum_{i=1}^{n}1_{X_{i}\in L_{b}\left(x\right)}},$$

where *L*_*b*_(*x*) contains neighbors of *x* in the *b*th tree. An ideal half-sampling estimator is then defined as

$$\begin{array}{l} {\hat{H}}_{n}^{HS}\left(x\right)={\left(\begin{array}{c} n\\ n/2 \end{array}\right)}^{-1}\underset{H:|H|=n/2}{\sum }{\left(E_{\Theta }\left[{\text{Ψ}}_{H}\left(x\right)\right]-E_{\Theta }\bar{\text{Ψ}}\left(x\right)\right)}^{2}\\ \;\bar{\text{Ψ}}\left(x\right)={\left(\begin{array}{c} n\\ n/2 \end{array}\right)}^{-1}\underset{H:|H|=n/2}{\sum }{\text{Ψ}}_{H}\left(x\right) \end{array}$$

where Θ is the randomness in building honest tree, including splitting data into random halves and randomness in selecting variables to split. ${\hat{H}}_{n}^{HS}\left(x\right)$ is similar to classic bootstrap estimator for the standard error, except that the sampling distribution for ${\hat{H}}_{n}^{HS}\left(x\right)$ is the half sampling distribution instead of the bootstrap sampling. Denote *E*_*ss*_ and *Var*_*ss*_ as the expectation and variance under the half sampling distribution, then ${\hat{H}}_{n}^{HS}\left(x\right)=Var_{ss}\left[E_{\Theta }\left[{\text{Ψ}}_{H}\left(x\right)\right]\right]$.

Since carrying out the full half-sampling computation and expectation with respect to Θ are impractical, Sexton and Laake (2009) pointed out that ${\hat{H}}_{n}^{HS}\left(x\right)$ can be efficiently approximated by the following law of total variance:

(5) $$\begin{array}{l} {\hat{H}}_{n}^{HS}\left(x\right)=Var_{ss}\left[E_{\Theta }\left[\frac{1}{M}\overset{M}{\underset{m=1}{\sum }}{\text{Ψ}}_{H,\Theta_{m}}\left(x\right)\right]\right]\\ \;=Var_{ss}\left[\frac{1}{M}\overset{M}{\underset{m=1}{\sum }}{\text{Ψ}}_{H,\Theta_{m}}\left(x\right)\right]\\ \;-E_{ss}\left[Var_{\Theta }\left[\frac{1}{M}\overset{M}{\underset{m=1}{\sum }}{\text{Ψ}}_{H,\Theta_{m}}\left(x\right)\right]\right] \end{array}$$

which leads to a Monte Carlo approximation of ${\hat{H}}_{n}^{HS}\left(x\right)$ by

(6) $$\begin{array}{l} {\hat{\sigma }}_{n}^{2}\left(x\right)={\hat{Var}}_{ss}\left[\frac{1}{M}\overset{M}{\underset{m=1}{\sum }}{\text{Ψ}}_{H,\Theta_{m}}\left(x\right)\right]\\ \;-{\hat{E}}_{ss}\left[{\hat{Var}}_{\Theta }\left[\frac{1}{M}\overset{M}{\underset{m=1}{\sum }}{\text{Ψ}}_{H,\Theta_{m}}\left(x\right)\right]\right]. \end{array}$$

In order to approximate random forest randomness quantity ${\hat{Var}}_{\Theta }$ and sampling randomness quantities ${\hat{Var}}_{ss},{\hat{E}}_{ss}$, we split *B* trees in *G* groups and each group has *l* trees, and the trees in the same group have the same half sample. The final consistent estimator ${\hat{\sigma }}_{n}^{2}\left(x\right)$ can be written as

$$\begin{array}{l} {\hat{\sigma }}_{n}^{2}\left(x\right)=\frac{1}{G-1}\overset{G}{\underset{g=1}{\sum }}{\left({\bar{\text{Ψ}}}_{g}\left(x\right)-\bar{\text{Ψ}}\left(x\right)\right)}^{2}\\ \;-\frac{1}{\left(l-1\right)}\frac{1}{B}\overset{G}{\underset{g=1}{\sum }}\overset{l}{\underset{i=1}{\sum }}{\left({\text{Ψ}}_{ig}\left(x\right)-{\bar{\text{Ψ}}}_{g}\left(x\right)\right)}^{2} \end{array}$$

where ${\bar{\text{Ψ}}}_{g}\left(x\right)=\frac{1}{l}\overset{l}{\underset{i=1}{\sum }}{\text{Ψ}}_{ig}\left(x\right)$, and $\bar{\text{Ψ}}\left(x\right)=\frac{1}{G}\overset{G}{\underset{g=1}{\sum }}{\bar{\text{Ψ}}}_{g}\left(x\right)$.

The following diagram summarizes the procedure of estimating the variance $\sigma_{n}^{2}\left(x\right)$.

$$\begin{array}{l} \sigma_{n}^{2}\left(x\right)\overset{\text{Asym.equivalent}}{\underset{\;}{\leftarrow }}s_{n}^{2}\left(x\right)\overset{\text{Half-Sampling estimator}}{\underset{\;}{\leftarrow }}\\ {\hat{H}}_{n}^{HS}\left(x\right)\overset{\text{Empirical estimator}}{\underset{\;}{\leftarrow }}{\hat{\sigma }}_{n}^{2}\left(x\right) \end{array}$$

where from left to right, the first arrow is based on Theorem 5 of Athey et al. (2018), the second arrow is based on half-sampling of Efron (1980), and the third arrow is supported by Equations (5) and (6) and the method of Sexton and Laake (2009).

## 4. Simulation Studies

We present simulations to evaluate the performance of the proposed method in finite sample setting. Two different models for the survival time are considered

- Model 1: $T=g^{-1}\left(\alpha_{0}+\overset{p}{\underset{i=1}{\sum }}\alpha_{i}X_{i}\right)+\epsilon$
- Model 2: $T=g^{-1}\left(\alpha_{0}+\overset{p}{\underset{i=1}{\sum }}\alpha_{i}X_{i}^{2}\right)+\epsilon$

where *X*_*i*1_, …, *X*_*ip*_ are independently generated from *Unif*(−1, 1), α_0_ = 5, α_1_ = α_2_ = 0.25 and α_*i*_ = 0 for *i* > 2, and ϵ ~ *N*(0, σ^2^). The variance σ^2^ is chosen to have proper signal-noise ratio (SNR),

$$\text{SNR}=\frac{Var\left(g^{-1}\left(\alpha_{0}+\sum_{i=1}^{p}\alpha_{i}X_{i}\right)\right)}{Var\left(\epsilon \right)}.$$

We generate the independent censoring time *C*_*i*_ from a Cox model with the following hazard λ = λ_*C*_ exp (*X*_1_ log 2) and λ_*C*_ is chosen to have a proper un-censoring rate. The link function *g* can have the following form

- Identity link: *g*^−1^(*x*) = *x*;
- Exp link: *g*^−1^(*x*) = exp(*x*);
- Log-exp link: *g*^−1^(*x*) = log(exp(*x*) + 1).

### 4.1. Evaluation of Coverage Probability of Predictions

To evaluate the asymptotic results in Theorem 1, we generate five training data sets and one testing data set with the same sample size. The coverage probability performance is evaluated on the testing data set with predictions and confidence intervals derived from 5 independent training data sets. More specifically, for each observation in the testing sample, we obtain the 95% confidence intervals and record how many times a hRMST observation in test sample is within five estimated 95% confidence intervals. The coverage probability of an observation is defined by the its proportion of being covered, and the overall coverage probability of the testing sample is defined by the average of coverage probability of each of its observation. We present the coverage probability results with sample size *n* = 1, 000, 2, 000, 5, 000 for Model 1, and *n* = 1, 000, 2, 000, 10, 000 for Model 2. By choosing the proper λ_*C*_, we control the un-censoring rate around 60–70% for different link functions: λ_*C*_ ~ 0.08 for Identity link and Log-exp link, and λ_*C*_ ~ 0.003 for Exp link. The truncation time *L* is chosen to make the truncation rate fall into 2%−5%. Specifically, *L* ~ 5.4 for Identity link and Log-exp link, and *L* ~ 220 for Exp link.

**Figure 2:**
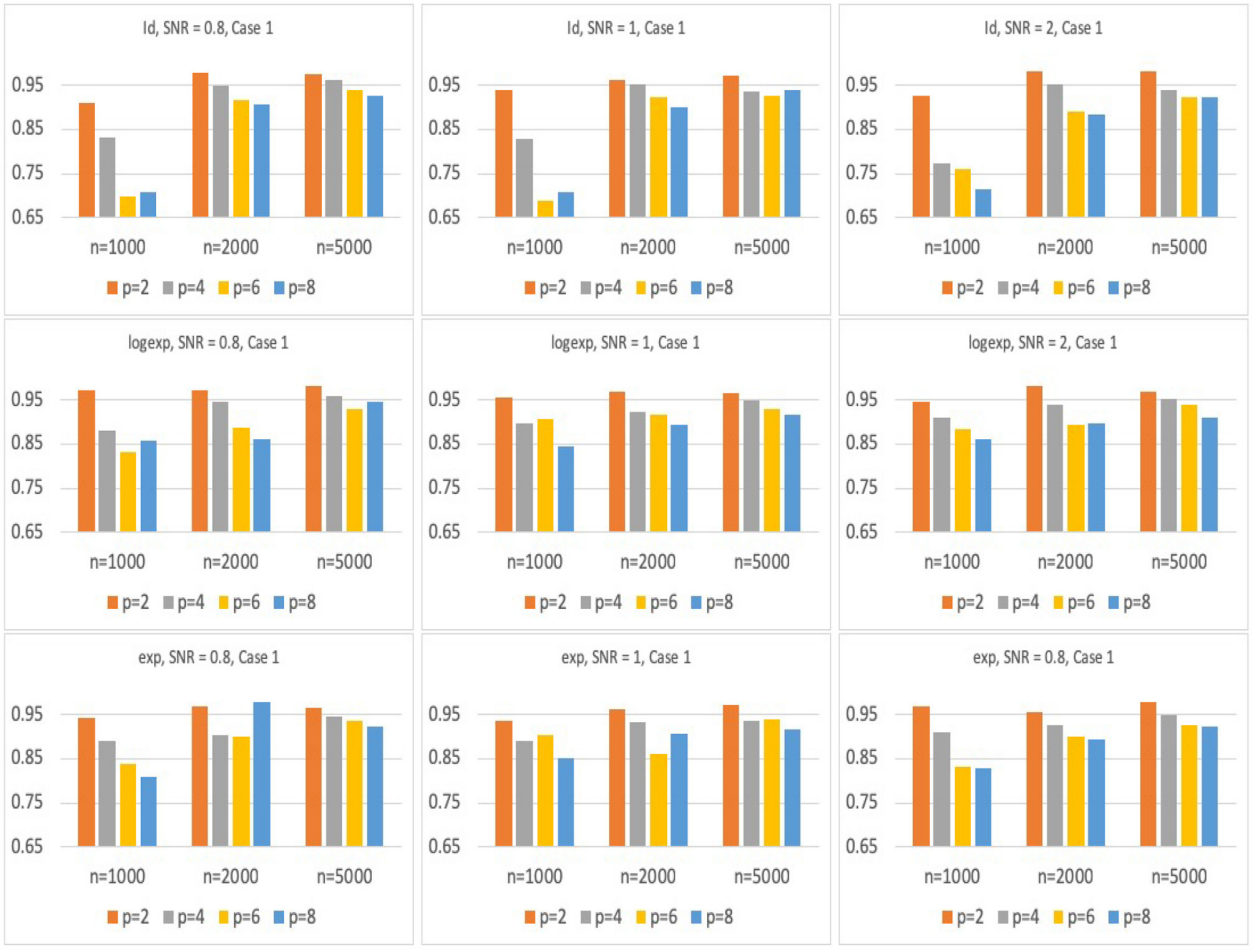
Simulation results of the coverage probability for Model 1 with three different link functions, sample size of *n* = 1, 000, 2, 000, 5, 000, and *p* = 2, 4, 6, 8. For each case, prediction coverage probability is calculated over the samples in the testing data set.

**Figure 3:**
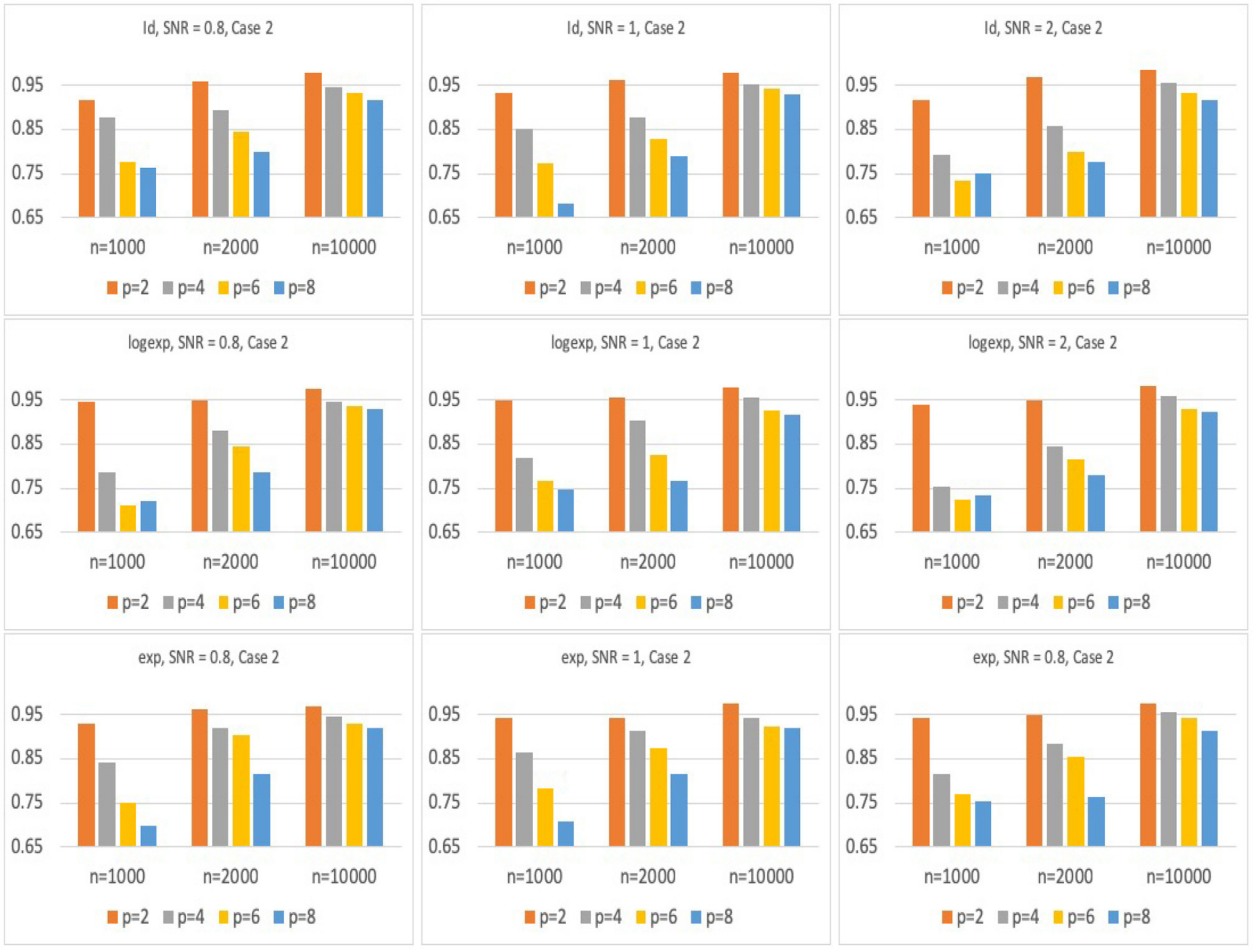
Simulation results of coverage probability for Model 2 with three different link functions, sample size of *n* = 1, 000, 2, 000, 10, 000, and *p* = 2, 4, 6, 8. For each case, prediction coverage probability is calculated over the samples in the testing data set.

Figures 2, 3 present the results for Model 1 and Model 2 under three different link functions. We see that the coverage probability approaches to nominal level 95% when the sample size gets larger. If *p* is smaller, the coverage probability is closer to 95%. This corresponds to the result of Theorem 3 in Wager and Athey (2015), which states that the rate of convergence of the bias of random forest estimator is $O\left(n^{\frac{K}{p}}\right)$ for some constant *K*. When the sample size *n* is fixed, bigger *p* leads to larger bias in the estimates of hRMST, and under-coverage of the confidence interval. On the other hand, when *p* is fixed, bigger *n* results in a smaller bias and leads to a better coverage of the confidence interval.

### 4.2. Comparison of Prediction Performance With Existing Methods

We compare our proposed method with several existing methods for hRMST estimation, including

- *Naive.km*: using Kaplan–Meier estimator for survival function and computing hRMST by Equation (1). Covariates are not adjusted.
- *Naive.Cox*: using proportational hazards estimator for the survival function and computing hRMST by Equation (1). The censoring distribution is assumed to follow the proportional hazards assumption.
- *Lu.method*: using some parametric forms of hRMST and computing hRMST by solving a weighted estimating equation. The censoring distribution is assumed to be independent of the covariates (Tian et al., 2014). We consider Identity link and Exp link in the simulations.
- *Wang.method*: using some parametric forms of hRMST and computing hRMST by solving a weighted estimating equation. The censoring distribution is assumed to follow the proportional hazards assumption. We consider Identity link and Exp link in the simulations (Wang and Schaubel, 2018).

We compare all these methods under Model 1 and Model 2, and use the Mean-Absolute-Error (MAE) and Rooted-Mean-Squared-Error (RMSE), introduced in Davison and Hinkley (1997), Tian et al. (2007), and Wang and Schaubel (2018), to measure the performance of these methods.

(7) $$\begin{array}{l} \text{MAE}=\frac{1}{n}\overset{n}{\underset{i=1}{\sum }}\frac{\delta_{i}^{L}}{1-\hat{G}\left(Z_{i}^{L}|X_{i}=x\right)}\left|Z_{i}^{L}-{\hat{\mu }}^{L}\left(X_{i}\right)\right|,\\ \text{RMSE}=\sqrt{\frac{1}{n}\overset{n}{\underset{i=1}{\sum }}\frac{\delta_{i}^{L}}{1-\hat{G}\left(Z_{i}^{L}|X_{i}=x\right)}[Z_{i}^{L}-{\hat{\mu }}^{L}\left(X_{i}\right){]}^{2}}. \end{array}$$

**Figure 4:**
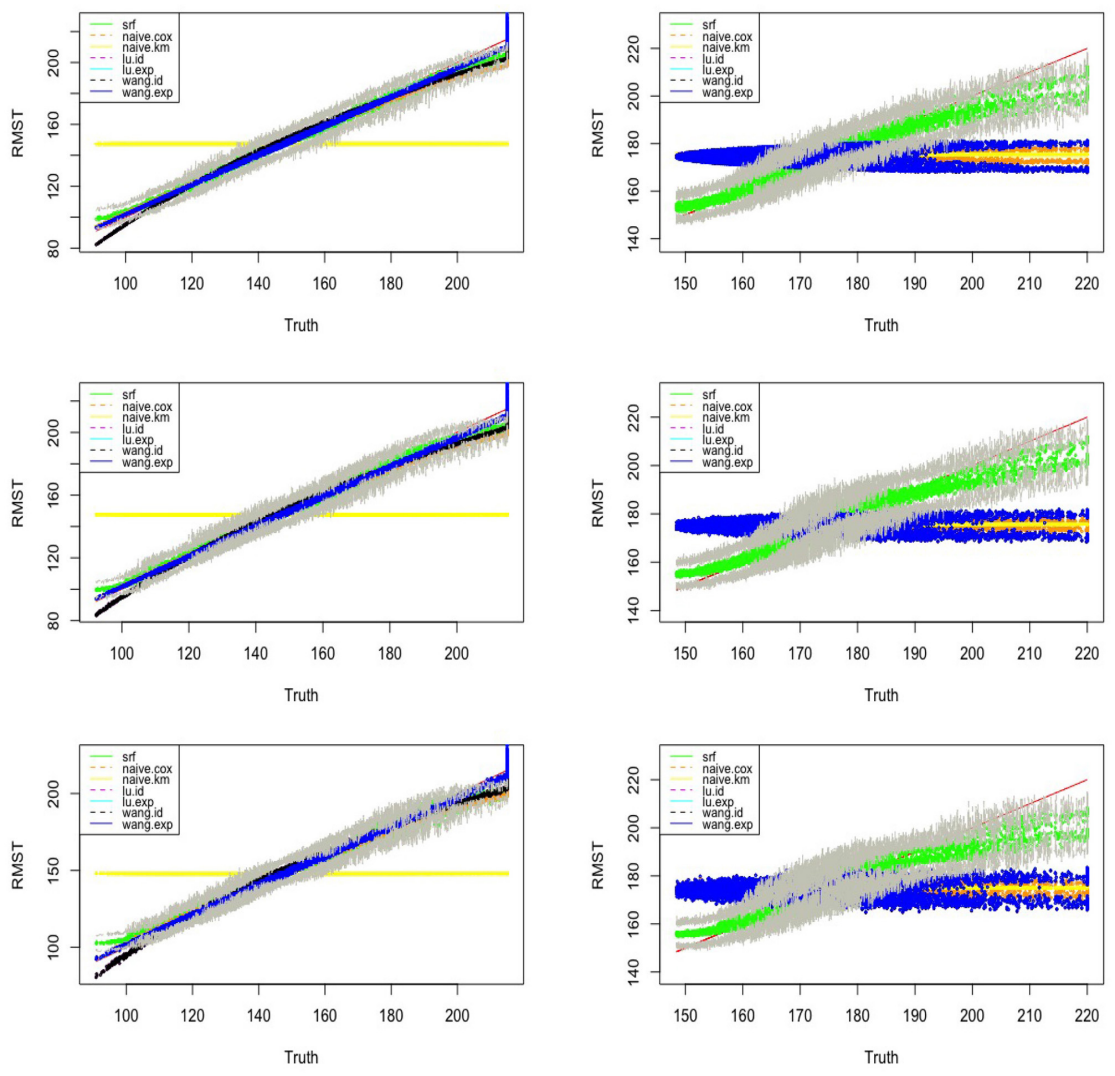
Estimated vs. the true RMST for Model 1 **(left)** and Model 2 **(right)** with exponential link function and the number of covariates *p* = 5, 10, 20 **(top–bottom)**. SRF, proposed random forest-bases estimator, and upper and lower bounds of the point-wise confidence intervals of the proposed random forest estimator are connected in the gray lines; Naive.km, estimate based on Kaplan–Meier estimator without adjusting for the covariates; Naive.Cox, Cox regression based estimator; Lu.id, method of Tian et al. (2014) with identity link; Lu.exp, method of Tian et al. (2014) with exponential link; Wang.id, method of Wang and Schaubel (2018) with identity link; Wang:exp, method of Wang and Schaubel (2018) with exponential link.

We set *n* = 3, 000, SNR = 0.3. For Identity link and Log-exp link, λ_*C*_ = 0.08, *L* = 5.3. For Exp link λ_*C*_ = 0.0026, *L* = 190. We calculate the MAE and RMSE for our method and four existing methods(both Lu.method and Wang.method have two link functions) under Model 1 and Model 2 and *p* = 5, 10, 20. Among all the considered models, our method in general has a better performance. As an example, Figure 4 visualizes the observed hRMST generated from Log-exp link and predicted hRMST from our method and Wang.method, showing that the random forest can give better predictions.

Tables 1, 2 show the MAE and RMSE for Model 1 and Model 2, respectively. For Model 1, the parametric models are correctly specified using the methods of Tian et al. (2014); Wang and Schaubel (2018), we expect that both methods perform well, and our method can have a comparable performance. For Model 2, our proposed method dominates all other methods. Increasing the number of non-predictive covariates does not have a big impact on the performance of our method.

**Table 1:** Comparison of Mean-Absolute-Error (MAE) and Rooted-Mean-Squared-Error (RMSE) for Model 1 with different link functions.

**p**	**SRF**	**Naive.Cox**	**Naive.km**	**Lu.id**	**Lu.exp**	**Wang.id**	**Wang.exp**
**Model 1: identity link, *n* = 3, 000, SNR = 0.3**							
5	0.1359	0.1371	0.2067	**0.1341**	0.1346	**0.1341**	0.1346
	0.1699	0.1695	0.2466	0.1687	0.1691	**0.1686**	0.1691
10	0.1396	0.1394	0.2108	**0.1371**	0.1377	**0.1371**	0.1376
	0.1721	0.1710	0.2497	0.1710	0.1715	**0.1709**	0.1714
20	0.1373	0.1372	0.2064	**0.1342**	0.1348	**0.1342**	0.1347
	0.1703	0.1693	0.2464	0.1686	0.1691	**0.1685**	0.1690
**Model 1: log-exp link, *n* = 3, 000, SNR = 0.3**							
5	0.1347	0.1359	0.2048	**0.1330**	0.1335	**0.1330**	0.1335
	0.1684	0.1680	0.2441	0.1673	0.1677	**0.1672**	0.1677
10	0.1384	0.1382	0.2088	**0.1359**	0.1366	**0.1359**	0.1365
	0.1706	**0.1695**	0.2472	**0.1695**	0.1701	**0.1695**	0.1699
20	0.1361	0.1360	0.2044	0.1331	0.1337	**0.1330**	0.1336
	0.1689	0.1679	0.2439	0.1672	0.1678	**0.1671**	0.1676
**Model 1: exp link, *n* = 3, 000, SNR = 0.3**							
5	24.724	25.398	33.688	24.496	24.723	**24.436**	24.709
	30.827	30.860	39.296	30.608	30.773	**30.577**	30.749
10	25.254	25.681	34.208	24.843	25.162	**24.812**	25.149
	31.085	31.052	39.621	30.869	31.076	**30.850**	31.048
20	24.878	25.260	33.587	24.390	24.679	**24.325**	24.651
	30.744	30.695	39.181	30.479	30.689	**30.438**	30.646

*The number of covariates p = 5, 10, 20, for each p, the first row is MAE, the second row is RMSE. SRF, proposed random forest-bases estimator; Naive.km, estimate based on Kaplan–Meier estimator without adjusting for the covariates; Naive.Cox, Cox regression based estimator; Lu.id, method of Tian et al. (2014) with identity link; Lu.exp, method of Tian et al. (2014) with exponential link; Wang.id, method of Wang and Schaubel (2018) with identity link; Wang:exp, method of Wang and Schaubel (2018) with exponential link*.

**Table 2:** Comparison of mean-absolute-error (MAE) and rooted-mean-squared-error (RMSE) for Model 2 with different link functions.

**p**	**SRF**	**Naive.Cox**	**Naive.km**	**Lu.id**	**Lu.exp**	**Wang.id**	**Wang.exp**
**Model 2: identity link, *n* = 3, 000, SNR = 0.3**							
5	**0.1218**	0.1386	0.1384	0.1388	0.1388	0.1382	0.1382
	**0.1498**	0.1658	0.1656	0.1660	0.1660	0.1656	0.1656
10	**0.1257**	0.1414	0.1412	0.1418	0.1418	0.1411	0.1411
	**0.1525**	0.1682	0.1679	0.1687	0.1687	0.1684	0.1684
20	**0.1239**	0.1390	0.1385	0.1393	0.1393	0.1387	0.1387
	**0.1507**	0.1662	0.1655	0.1667	0.1667	0.1663	0.1663
**Model 2: log-exp link, *n* = 3, 000, SNR = 0.3**							
5	**0.1201**	0.1366	0.1364	0.1368	0.1368	0.1362	0.1362
	**0.1479**	0.1635	0.1633	0.1637	0.1637	0.1634	0.1634
10	**0.1240**	0.1395	0.1393	0.1399	0.1399	0.1392	0.1392
	**0.1506**	0.1660	0.1657	0.1664	0.1664	0.1661	0.1661
20	**0.1222**	0.1371	0.1366	0.1374	0.1374	0.1368	0.1368
	**0.1487**	0.1640	0.1633	0.1645	0.1645	0.1641	0.1641
**Model 2: exp link, *n* = 3, 000, SNR = 0.3**							
5	**21.030**	23.794	23.733	23.915	23.911	23.542	23.541
	**25.984**	28.185	28.135	28.297	28.292	28.126	28.125
10	**21.641**	24.165	24.127	24.322	24.319	23.928	23.928
	**26.357**	28.475	28.430	28.618	28.614	28.473	28.472
20	**21.368**	23.802	23.712	23.956	23.952	23.571	23.571
	**26.071**	28.216	28.102	28.379	28.375	28.208	28.207

*The number of covariates p = 5, 10, 20, for each p, the first row is MAE, the second row is RMSE. SRF, proposed random forest-bases estimator; Naive.km, estimate based on Kaplan–Meier estimator without adjusting for the covariates; Naive.Cox, Cox regression based estimator; Lu.id, method of Tian et al. (2014) with identity link; Lu.exp, method of Tian et al. (2014) with exponential link; Wang.id, method of Wang and Schaubel (2018) with identity link; Wang:exp, method of Wang and Schaubel (2018) with exponential link*.

When the censoring distribution does not follow PH assumption, we may expect a difference in the prediction performance because of the bias of IPCW from mis-specification. To check whether our method can still outperform the existing methods, we conduct additional numerical studies. In particular, we simulate the censoring time from the following gamma distributions

$$C\sim \Gamma \left(\alpha ,\beta \right),\beta =\frac{1}{\lambda_{C}\exp\left(X_{1}\log2\right)},\text{ and }\alpha \in \left\{0.5,1.5\right\}$$

When α = 1, the gamma distribution degenerates to the exponential distribution we used for Tables 1, 2. Tables 3, 4 show the MAE and RMSE for Model 1 and Model 2 when α = 0.5, and Tables 5, 6 show the MAE and RMSE for Model 1 and Model 2 when α = 1.5. Results of α ∈ {0.5, 1.5} are not very different from the results of α = 1. Under Model 1, our method performs comparably well as methods of Tian et al. (2014); Wang and Schaubel (2018), and it dominates the others under Model 2. When feature dimension is low(*p* = 5), the error metrics of our method when α = 1 are in general lower than the error metrics when α = 0.5, 1.5 for both Model 1 and Model 2. The additional errors can be regarded as the bias induced from the violation of PH assumption of the censoring distribution. When feature dimension is high(*p* = 10, 20), bias from large *p* may dominate the bias from the violation of PH assumption of the censoring distribution.

**Table 3:** Comparison of Mean-Absolute-Error (MAE) and Rooted-Mean-Squared-Error (RMSE) for Model 1 with different link functions and the censoring distribution is mis-specified with α = 0.5.

**p**	**SRF**	**Naive.Cox**	**Naive.km**	**Lu.id**	**Lu.exp**	**Wang.id**	**Wang.exp**
**Model 1: identity link, *n* = 3, 000, SNR = 0.3**							
5	0.1361	0.1353	0.2051	0.1337	0.1344	**0.1336**	0.1342
	0.1706	**0.1681**	0.2457	0.1687	0.1693	0.1685	0.1690
10	0.1444	0.1430	0.2160	**0.1402**	0.1408	0.1403	0.1408
	0.1755	0.1732	0.2523	0.1726	0.1731	**0.1725**	0.1730
20	0.1392	0.1372	0.2078	**0.1345**	0.1351	**0.1345**	0.1351
	0.1723	0.1699	0.2484	0.1694	0.1700	**0.1692**	0.1698
**Model 1: log-exp link, *n* = 3, 000, SNR = 0.3**							
5	0.1348	0.1341	0.2032	0.1325	0.1333	**0.1324**	0.1330
	0.1691	**0.1667**	0.2432	0.1673	0.1679	0.1671	0.1676
10	0.1431	0.1418	0.2139	**0.1390**	0.1396	0.1391	0.1396
	0.1740	0.1718	0.2497	0.1712	0.1717	**0.1711**	0.1716
20	0.1380	0.1360	0.2060	0.1335	0.1341	**0.1334**	0.1340
	0.1708	0.1685	0.2460	0.1681	0.1687	**0.1679**	0.1685
**Model 1: exp link, *n* = 3, 000, SNR = 0.3**							
5	24.906	25.157	33.628	24.471	24.826	**24.427**	24.784
	30.984	30.687	39.205	30.609	30.852	**30.591**	30.800
10	26.381	26.553	35.410	25.738	26.015	**25.678**	25.996
	31.799	31.593	40.265	31.403	31.607	**31.373**	31.574
20	25.096	25.145	33.418	24.461	24.741	**24.365**	24.680
	30.940	30.746	39.152	30.609	30.831	**30.551**	30.759

*The number of covariates p = 5, 10, 20, for each p, the first row is MAE, the second row is RMSE. SRF, proposed random forest-bases estimator; Naive.km, estimate based on Kaplan–Meier estimator without adjusting for the covariates; Naive.Cox, Cox regression based estimator; Lu.id, method of Tian et al. (2014) with identity link; Lu.exp, method of Tian et al. (2014) with exponential link; Wang.id, method of Wang and Schaubel (2018) with identity link; Wang:exp, method of Wang and Schaubel (2018) with exponential link*.

**Table 4:** Comparison of Mean-Absolute-Error (MAE) and Rooted-Mean-Squared-Error (RMSE) for Model 2 with different link functions and the censoring distribution is mis-specificed with α = 0.5.

**p**	**SRF**	**Naive.Cox**	**Naive.km**	**Lu.id**	**Lu.exp**	**Wang.id**	**Wang.exp**
**Model 1: identity link, *n* = 3, 000, SNR = 0.3**							
5	**0.1230**	0.1378	0.1374	0.1385	0.1385	0.1377	0.1377
	**0.1514**	0.1657	0.1653	0.1663	0.1663	0.1658	0.1658
10	**0.1310**	0.1450	0.1442	0.1457	0.1457	0.1447	0.1447
	**0.1562**	0.1704	0.1695	0.1712	0.1712	0.1704	0.1704
20	**0.1262**	0.1394	0.1384	0.1403	0.1403	0.1392	0.1392
	**0.1533**	0.1668	0.1657	0.1681	0.1681	0.1673	0.1673
**Model 1: log-exp link, *n* = 3, 000, SNR = 0.3**							
5	**0.1213**	0.1359	0.1355	0.1365	0.1365	0.1358	0.1358
	**0.1494**	0.1634	0.1630	0.1640	0.1640	0.1636	0.1636
10	**0.1292**	0.1430	0.1422	0.1437	0.1437	0.1427	0.1427
	**0.1543**	0.1681	0.1673	0.1689	0.1689	0.1681	0.1681
20	**0.1244**	0.1374	0.1364	0.1383	0.1383	0.1372	0.1372
	**0.1512**	0.1645	0.1634	0.1658	0.1658	0.1650	0.1650
**Model 1: exp link, *n* = 3, 000, SNR = 0.3**							
5	**21.270**	23.793	23.697	24.016	24.009	23.535	23.534
	**26.187**	28.147	28.075	28.329	28.322	28.133	28.132
10	**22.824**	25.159	24.946	25.408	25.399	24.843	24.842
	**27.067**	29.009	28.823	29.239	29.227	28.945	28.943
20	**21.832**	23.896	23.708	24.188	24.177	23.698	23.697
	**26.635**	28.417	28.221	28.753	28.740	28.499	28.499

*The number of covariates p = 5, 10, 20, for each p, the first row is MAE, the second row is RMSE. SRF, proposed random forest-bases estimator; Naive.km, estimate based on Kaplan–Meier estimator without adjusting for the covariates; Naive.Cox, Cox regression based estimator; Lu.id, method of Tian et al. (2014) with identity link; Lu.exp, method of Tian et al. (2014) with exponential link; Wang.id, method of Wang and Schaubel (2018) with identity link; Wang:exp, method of Wang and Schaubel (2018) with exponential link*.

**Table 5:** Comparison of Mean-Absolute-Error (MAE) and Rooted-Mean-Squared-Error (RMSE) for Model 1 with different link functions and the censoring distribution is mis-specificed with α = 1.5.

**p**	**SRF**	**Naive.Cox**	**Naive.km**	**Lu.id**	**Lu.exp**	**Wang.id**	**Wang.exp**
**Model 1: identity link, *n* = 3, 000, SNR = 0.3**							
5	0.1363	0.1378	0.2067	**0.1352**	0.1357	**0.1352**	0.1357
	0.1701	0.1702	0.2467	**0.1697**	0.1702	**0.1697**	0.1702
10	0.1376	0.1385	0.2073	**0.1358**	0.1363	**0.1358**	0.1363
	0.1709	0.1706	0.2472	**0.1699**	0.1704	**0.1699**	0.1704
20	0.1371	0.1371	0.2062	**0.1341**	0.1347	0.1342	0.1347
	0.1698	0.1691	0.2464	**0.1682**	0.1688	**0.1682**	0.1688
**Model 1: log-exp link, *n* = 3, 000, SNR = 0.3**							
5	0.1350	0.1366	0.2046	**0.1340**	0.1345	**0.1340**	0.1345
	0.1686	0.1687	0.2441	**0.1683**	0.1688	**0.1683**	0.1688
10	0.1363	0.1373	0.2053	**0.1346**	0.1352	0.1347	0.1352
	0.1695	0.1692	0.2447	**0.1685**	0.1690	**0.1685**	0.1690
20	0.1359	0.1359	0.2043	**0.1330**	0.1335	**0.1330**	0.1336
	0.1683	0.1677	0.2439	**0.1669**	0.1674	**0.1669**	0.1674
**Model 1: exp link, *n* = 3, 000, SNR = 0.3**							
5	24.537	25.171	33.190	24.322	24.601	**24.304**	24.600
	30.701	30.750	38.999	30.549	30.735	**30.532**	30.715
10	24.802	25.317	33.359	24.468	24.743	**24.445**	24.744
	30.798	30.832	39.142	30.577	30.757	**30.560**	30.742
20	24.852	25.188	33.406	24.300	24.567	**24.272**	24.570
	30.732	30.654	39.103	30.384	30.583	**30.371**	30.576

*The number of covariates p = 5, 10, 20, for each p, the first row is MAE, the second row is RMSE. SRF, proposed random forest-bases estimator; Naive.km, estimate based on Kaplan–Meier estimator without adjusting for the covariates; Naive.Cox, Cox regression based estimator; Lu.id, method of Tian et al. (2014) with identity link; Lu.exp, method of Tian et al. (2014) with exponential link; Wang.id, method of Wang and Schaubel (2018) with identity link; Wang:exp, method of Wang and Schaubel (2018) with exponential link*.

**Table 6:** Comparison of Mean-Absolute-Error (MAE) and Rooted-Mean-Squared-Error (RMSE) for Model 2 with different link functions and the censoring distribution is mis-specificed with α = 1.5.

**p**	**SRF**	**Naive.Cox**	**Naive.km**	**Lu.id**	**Lu.exp**	**Wang.id**	**Wang.exp**
**Model 1: identity link, *n* = 3, 000, SNR = 0.3**							
5	**0.1227**	0.1396	0.1395	0.1397	0.1397	0.1394	0.1394
	**0.1507**	0.1666	0.1664	0.1668	0.1668	0.1666	0.1666
10	**0.1241**	0.1391	0.1389	0.1393	0.1393	0.1390	0.1390
	**0.1514**	0.1667	0.1664	0.1669	0.1669	0.1668	0.1668
20	**0.1232**	0.1390	0.1386	0.1393	0.1393	0.1389	0.1389
	**0.1499**	0.1659	0.1654	0.1663	0.1663	0.1661	0.1661
**Model 1: log-exp link, *n* = 3, 000, SNR = 0.3**							
5	**0.1210**	0.1376	0.1375	0.1378	0.1378	0.1374	0.1374
	**0.1487**	0.1643	0.1642	0.1645	0.1645	0.1643	0.1643
10	**0.1224**	0.1372	0.1370	0.1374	0.1374	0.1371	0.1371
	**0.1494**	0.1644	0.1642	0.1646	0.1646	0.1645	0.1645
20	**0.1215**	0.1371	0.1368	0.1374	0.1374	0.1370	0.1370
	**0.1480**	0.1637	0.1632	0.1641	0.1641	0.1638	0.1638
**Model 1: exp link, *n* = 3, 000, SNR = 0.3**							
5	**21.071**	23.719	23.699	23.787	23.785	23.581	23.580
	**26.092**	28.241	28.217	28.313	28.311	28.238	28.238
10	**21.334**	23.649	23.612	23.711	23.710	23.524	23.524
	**26.159**	28.231	28.186	28.283	28.281	28.224	28.224
20	**21.176**	23.629	23.571	23.748	23.745	23.492	23.492
	**25.893**	28.077	27.993	28.208	28.204	28.085	28.085

*The number of covariates p = 5, 10, 20, for each p, the first row is MAE, the second row is RMSE. SRF, proposed random forest-bases estimator; Naive.km, estimate based on Kaplan–Meier estimator without adjusting for the covariates; Naive.Cox, Cox regression based estimator; Lu.id, method of Tian et al. (2014) with identity link; Lu.exp, method of Tian et al. (2014) with exponential link; Wang.id, method of Wang and Schaubel (2018) with identity link; Wang:exp, method of Wang and Schaubel (2018) with exponential link*.

## 5. Application to the TCGA Ovarian Cancer Data Set

We apply the proposed method to The Cancer Genome Atlas (TCGA) ovarian cancer functional proteomics data set (Akbani et al., 2015) that is publicly available (http://gdac.broadinstitute.org). The data sets include proteomic characterization of tumors using reverse-phase protein arrays (RPPA). Specifically, Akbani et al. (2015) reported an RPPA-based proteomic analysis using 195 high-quality antibodies that target total, cleaved, acetylated and phosphorylated forms of proteins in 412 high-grade serous ovarian cystadenocarcinoma (OVCA) samples. The function space covered by the antibodies used in the RPPA analysis emcompasses major functional and signaling pathways of relevance to human cancer, including proliferation, DNA damage, polarity, vesicle function, EMT, invasiveness, hormone signaling, apoptosis, metabolism, immunological, and stromal function as well as transmembrane receptors, integrin, TGFβ, LKB1/AMPK, TSC/mTOR, PI3K/Akt, Ras/MAPK, Hippo, Notch, and Wnt/beta-catenin signaling (Akbani et al., 2015).

After removing a few samples with missing data, the final data set includes 407 OVCA samples with a mean/median follow-up of 3.20/2.79 years and a total of 242 deaths and 40% censoring. To assess how different methods predict the hRMST, we performed the following cross-validation analysis. For a given *L*, we did 10-fold cross-validation on the data set. For each training data set in the cross-validation, we perform a univariate analysis to select top 5 most significant features based on univariate Cox regression analysis. We then estimate the hRMST on the test set using the training data sets with these 5 features as the predictors. We apply 7 different methods, including estimate based on the KM estimator, estimate based on the Cox model, the method of Tian et al. (2014) and the method of Wang and Schaubel (2018). We report the average of MAE and RMSE on the samples in the testing sets over the 10-fold cross-validation.

**Figure 5:**
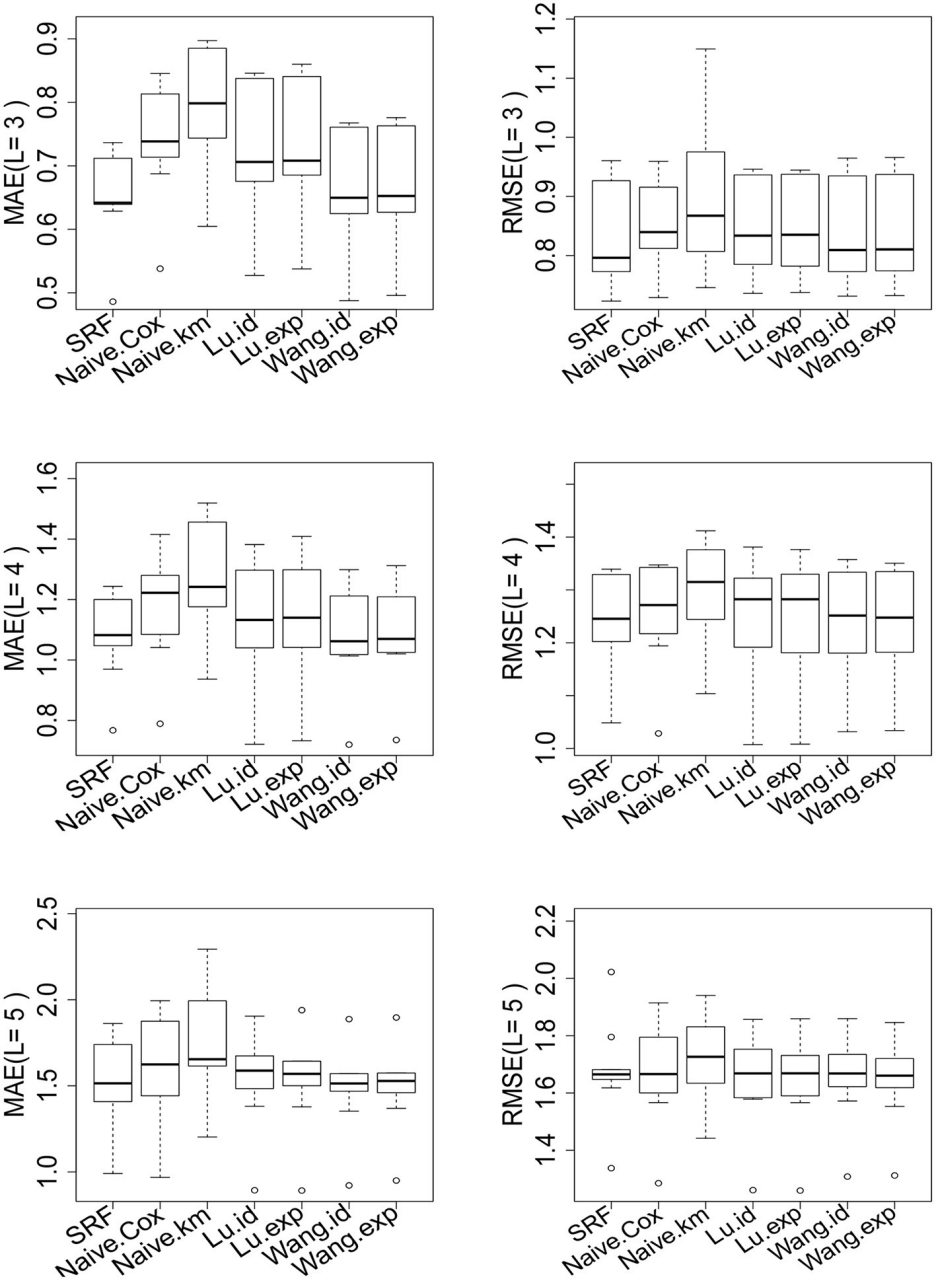
Performance of the proposed random forest estimator compared with other methods for *L* = 3, 4, 5. The left penal is the MAE across of 10-fold cross-validation. The right panel is the RMSE across of 10-fold cross-validation. SRF, proposed random forest estimator; Naive.km, estimate based on Kaplan–Meier estimator without adjusting for the covariates; Naive.Cox, Cox regression based estimator; Lu.id, method of Tian et al. (2014) with identity link; Lu.exp, method of Tian et al. (2014) with exponential link; Wang.id method of Wang and Schaubel (2018) with identity link; Wang:exp, method of Wang and Schaubel (2018) with exponential link.

The results are shown in Table 7 and Figure 5 for *L* = 3, 4, 5 (see Supplementary Material for *L* = 6, 7, 8). There are 45.9, 31.2, 19.4, 11.8, 8.1, 4.4% of the observations larger than *L* for *L* = 3, 4, 5, 6, 7, 8 correspondingly. For different choices of *L*, our proposed random forest based method dominates the other methods in MAE and RMSE. The methods of Tian et al. (2014) and Wang and Schaubel (2018) are based on parametric form of hRMST. Cox model is heavily dependent on the proportional hazard assumption, and the Kaplan–Meier approach does not take the covariates into account. We also notice that the method of Wang and Schaubel (2018) always performs better than the method of Tian et al. (2014), possibly due to the fact that the censoring mechanism in the data depends on the covariates.

**Table 7:** Performance of the proposed random forest estimator compared with other methods for *L* = 3, 4, 5.

**L**	**SRF**	**Naive.Cox**	**Naive.km**	**Lu.id**	**Lu.exp**	**Wang.id**	**Wang.exp**
3	**0.6879**	0.9247	0.9463	0.9266	0.9355	0.7630	0.7721
	**0.8258**	0.8925	0.8967	0.8966	0.8983	0.8438	0.8455
4	**1.2033**	1.5450	1.5686	1.5704	1.5777	1.2862	1.3044
	**1.2403**	1.3597	1.3648	1.3830	1.3817	1.2719	1.2752
5	**1.7479**	2.2107	2.2395	2.2467	2.2306	1.8251	1.8540
	**1.6761**	1.8594	1.8655	1.8989	1.8858	1.7168	1.7193

*The first row is MAE, the second row is RMSE. SRF, proposed random forest estimator; Naive.km, estimate based on Kaplan–Meier estimator without adjusting for the covariates; Naive.Cox, Cox regression based estimator; Lu.id, method of Tian et al. (2014) with identity link; Lu.exp, method of Tian et al. (2014) with exponential link; Wang.id, method of Wang and Schaubel (2018) with identity link; Wang:exp, method of Wang and Schaubel (2018) with exponential link*.

## 6. Discussion

In this paper, we have developed a non-parametric random forest-based method for estimation of hRMST. Compared with traditional Cox model, which gets hRMST estimates by transforming the estimated hazard functions, directly modeling hRMST would be more preferable for computation and feature importance analysis. The proposed estimator can relax the parametric assumptions imposed on the survival time used in Tian et al. (2014) and Wang and Schaubel (2018), and can achieve better prediction performance. We have derived the asymptotic distribution of the random forest estimator using IPCW approach, and presented a procedure based on bags of little bootstraps to obtain the variance of the estimator. Our simulation results and analysis of TCGA data sets have shown promising performance in predicting hRMST as compared to the other available methods, even when the dimension is high and the covariates include irrelevant variables. The method is implemented by R and C++, and is available at https://github.com/lmy1019/SRF.

The proposed method can be used to estimate the heterogeneous treatment effects in randomized clinical trials when the outcome is censored. One can simply apply the method separately to the treated group and the placebo group and take the difference. However, for the observational studies, one needs to account for the fact that the treatment assignments might not be completely at random. Wager and Athey (2015) developed a non-parametric causal forest for estimating heterogeneous treatment effects that extends Breiman's random forest algorithm. In the potential outcomes framework with non-confounding, they showed that causal forest are pointwise consistent for the true treatment effect and have an asymptotically Gaussian and centered sampling distribution. For the observational studies with censored survival outcomes, it is also possible to combine the methods proposed here and the method of Wager and Athey (2015) in order to estimate the treatment effect on the restricted mean survival time.

The proposed methods can also be extended to take into account possible competing risk. This can be done by introducing an additional inverse probability weight (IPCW) to differentiate the non-informative censoring and competing risk censoring. In this case, the estimation equation ψ function with covariates history $\tilde{X}=\tilde{x}$ under true *G*_*C*_ and *G*_*R*_ becomes

(8) $$\begin{array}{l} {\tilde{\psi }}_{\mu }\left(\tilde{x},Z^{L},\delta^{L}\right)=\frac{1}{1-G_{C}\left(Z^{L}|X=x\right)}\frac{1}{1-G_{R}\left(Z^{L}|\tilde{X}=\tilde{x}\right)}\\ \;\delta^{L}\left(Z^{L}-\mu \right), \end{array}$$

where under competing risk scenario, $\delta^{L}=1_{\left\{T\wedge L\leq C\wedge R\right\}}$. The method proposed in this paper can be automatically adapted to the competing risk case and the asymptotic normality result can be derived similarly.

## Data Availability Statement

The original contributions presented in the study are included in the article/Supplementary Materials, further inquiries can be directed to the corresponding author/s.

## Author Contributions

ML and HL developed the ideas and the methods together, analyzed the real data sets, and wrote the manuscript. ML implemented the methods and performed the numerical analysis. All authors contributed to the article and approved the submitted version.

## Conflict of Interest

The authors declare that the research was conducted in the absence of any commercial or financial relationships that could be construed as a potential conflict of interest.
