# Supplementary material for: Estimation of Heterogeneous Restricted Mean Survival Time Using Random Forest
Source: Front Genet. 2021 Jan 7;11:587378. doi: 10.3389/fgene.2020.587378 (PMC7873855; doi:10.3389/fgene.2020.587378)
Supplement: Supplementary file 1 [file Data_Sheet_1.PDF]

Supplementary Material

1 PROOFS

1.1 Supporting Results

Before we prove the main theorm, we first define some auxiliry quantities under true IPCW.

$$\tilde{\mu}^L(x) = \sum_{i=1}^{n} \frac{\alpha_i(x) w_i Z_i^L}{\sum_{i=1}^{n} \alpha_i(x) w_i},$$
$$\tilde{\psi}_{\mu^L(x)}(X, Z^L, \delta^L) = w_i(Z^L - \mu^L(x))$$
$$\tilde{\Psi}_{\mathcal{H}} = \frac{1}{|S_{\mathcal{H}}|} \sum_{b \in S_{\mathcal{H}}} \frac{\sum_{i=1}^{n} 1_{x_i \in L_b(x)} \tilde{\psi}_{\hat{\mu}^L(x)}(X_i, Z_i^L, \delta_i^L)}{\sum_{i=1}^{n} 1_{x_i \in L_b(x)}},$$
$$\tilde{\sigma}_n(x) = \frac{1}{G-1} \sum_{g=1}^{G} (\bar{\tilde{\Psi}}_g - \bar{\tilde{\Psi}})^2 - \frac{1}{(l-1)} \frac{1}{B} \sum_{g=1}^{G} \sum_{i=1}^{l} (\tilde{\Psi}_{ig} - \bar{\tilde{\Psi}}_g)^2.$$

LEMMA 1. $\tilde{\psi}_\mu(X_i, Z_i^L, \delta_i^L)$ *satisfies Assumption 1-6 in Athey et al. (2018)*

PROOF OF LEMMA 1. Let $M_\mu(x) = E[\tilde{\psi}_\mu(X, Z^L, \delta^L)|X = x] = \mu^L(x) - \mu$. For the first assumption, $M_\mu(x)$ is Lipschitz continuous w.r.t x by our Assumption 1. For the second assumption, $M_\mu(x)$ is twice continuously differentiable in μ with uniformly bounded second derivative, and $V(x) := \frac{d}{d\mu} M_\mu(x)|_{\mu=\mu^L(x)} = -1$, which is invertible. For the third assumption,

$$\begin{aligned}
\gamma(\mu, \mu') &= \sup\{||Var[\tilde{\psi}_\mu(X_i, Z_i^L, \delta_i^L) - \tilde{\psi}_{\mu'}(X_i, Z_i^L, \delta_i^L)|X_i = x]||_F\} \\
&= \sup_x\{||Var[w_i(\mu - \mu')|X_i = x]||_F\} \\
&\leq \sup_x ||\mu - \mu'||_2^2 E[w_i^2|X_i = x] \\
&\leq \frac{1}{\epsilon_L} ||\mu - \mu'||_2^2
\end{aligned}$$

by Assumption 2. For fourth assumption, $\tilde{\psi}_\mu(X, Z^L, \delta^L)$ is itself Lipschitz w.r.t μ by Assumption 2. For fifth assumption, for any $\alpha_i, \sum_{i=1}^{n} \alpha_i = 1$, there exists $\mu^\alpha = \frac{\sum_{i=1}^{n} \alpha_i w_i Z_i^L}{\sum_{i=1}^{n} \alpha_i w_i}$, such that $|| \sum_{i=1}^{n} \alpha_i \tilde{\psi}_{\mu^\alpha}(X_i, Z_i^L, \delta_i^L)||_2 = 0$. For the last assumption, $\tilde{\psi}_\mu(X_i, Z_i^L, \delta_i^L)$ is the negative subgradient of a convex function w.r.t μ, because $\tilde{\psi}_\mu(X_i, Z_i^L, \delta_i^L) = -\frac{d}{d\mu}\left(\frac{1}{2} w_i (Z_i^L - \mu)^2\right)$, and $M_\mu(x)$ is the negative gradient of a strongly convex function w.r.t μ, because $M_\mu(x) = -\frac{d}{d\mu} \frac{1}{2}(\mu^L(x) - \mu)^2$.

LEMMA 2 (Athey et al. (2018) Theorem 5). *Under Assumption 1, 2, 3 and the forest is trained according to Specification 1 (Athey et al., 2018), then there exists a sequence of* $\bar{\sigma}_n(x)$*,*

$$\frac{\tilde{\mu}^L(x) - \mu^L(x)}{\bar{\sigma}_n(x)} \rightarrow_d N(0,1), \text{ where } \bar{\sigma}_n^2(x) = polylog(n/s)^{-1}(s/n)$$

if subsampling size $\beta_{\min} = 1 - \left(1 + \frac{\pi^{-1}\left(\log(\omega^{-1})\right)}{\log\left((1-\omega)^{-1}\right)}\right)^{-1}$. $ploy(x)$ *stands for a function that increases at most in polynomial order with* x*.*

PROOF OF LEMMA.2. We only need to show $Var[\tilde{\psi}_{\mu^L(x)}(X_i, Z_i^L, \delta_i^L)|X = x] > 0$.

$$\begin{aligned} & Var[\tilde{\psi}_{\mu^L(x)}(X_i, Z_i^L, \delta_i^L)|X = x] \\ = \; & E[\frac{(T \wedge L - \mu^L(x))^2}{1 - G(T^L|X)}|X = x] - (E[T \wedge L - \mu^L(x)|X = x])^2 \\ = \; & E[\frac{(T \wedge L - \mu^L(x))^2}{1 - G(T \wedge L|X)}|X = x] \geq Var(T \wedge L|X = x) > 0, \end{aligned}$$

where the last inequality is from Assumption 3.

Lemma 2 implies that the second term in the right hand side of Eq. (S6) is asymptotically normal. However, G is not known beforehand. In practice, Tian et al. (2014) estimates $G(\cdot)$ by KM estimator under the assumption that $G(\cdot)$ doesn't depend on covariates. Wang and Schaubel (2018) estimates $G(\cdot|\cdot)$ by Cox-model. We show that $\hat{\mu}^L(x)$ is also asymptotically normal even if $\hat{G}$ is estimated from the Cox model using the training set. That is, the first term in the right hand side of Eq. (S6) is $o_p(1)$. Below is a useful lemma quantifying the convergence rate of $\hat{w}(t)$ estimated from the Cox model.

LEMMA 3 (Wang and Schaubel (2018) Theorem 2 in Section A.5). *Under Assumption (4) and proper regularity conditions*

$$\sqrt{n}\left(\frac{1}{1 - G(t|X_i)} - \frac{1}{1 - \hat{G}(t|X_i)}\right) =$$

$$\frac{1}{\sqrt{n}} \frac{1}{1 - G(t|X_i)} \left(D_i(t)'[\Omega(\beta_C)]^{-1} \sum_{j=1}^{n} U_j(\beta_C) + \sum_{j=1}^{n} J_{ij}(t) \right) + o_p(1),$$

where

$$D_i(t) = \int_0^t (X_i - \bar{x}(u; \beta_C))\lambda_i^C(u)du,$$

$$U_i(\beta) = \int_0^\tau (X_i - \bar{x}(u, \beta))dM_i^C(u),$$

with $dM_i^C(t) = dN_i^C(t) - R_i(t)\lambda_i^C(t)dt$*.*

$$J_{ij}^C(t) = \int_0^t \exp(\beta_C' X_i) R_i(u) [r^{(0)}(t, \beta_C)]^{-1} dM_j^C(u).$$

Next Lemma shows that the scaled estimators with true IPCW and estimated IPCW are asymptotic equivalent.

LEMMA 4. *Under the assumptions of Lemma 2, 3*

$$\frac{\hat{\mu}^L(x) - \tilde{\mu}^L(x)}{\bar{\sigma}_n(x)} = o_p(1)$$

PROOF OF LEMMA.4. Note that $\tilde{\mu}^L(x) - \hat{\mu}^L(x) = \frac{a_n}{b_n} - \frac{a_n'}{b_n'}$, where

$$a_n = \sum_{i=1}^{n} \alpha_i(x) w_i Z_i^L, a_n' = \sum_{i=1}^{n} \alpha_i(x) \hat{w}_i Z_i^L, b_n = \sum_{i=1}^{n} \alpha_i(x) w_i, b_n' = \sum_{i=1}^{n} \alpha_i(x) \hat{w}_i$$

We then separate the proof into three parts.

$$|a_n - a_n'| = O_p(\frac{1}{\sqrt{n}}), |b_n - b_n'| = O_p(\frac{1}{\sqrt{n}}) \tag{S1}$$

$$\frac{|b_n - b_n'|}{b_n'} = O_p(\frac{1}{\sqrt{n}}), \frac{|a_n - a_n'|}{b_n'} = O_p(\frac{1}{\sqrt{n}}) \tag{S2}$$

$$\tilde{\mu}^L(x) - \hat{\mu}^L(x) = \frac{a_n}{b_n} - \frac{a_n'}{b_n'} = O_p(\frac{1}{\sqrt{n}}) \tag{S3}$$

To prove (S1),

$$\begin{aligned} |b_n - b_n'| &= |\sum_{i=1}^{n} \alpha_i(x)(w_i - \hat{w}_i)| \\ =& |\sum_{i=1}^{n} \alpha_i(x) \frac{1}{n} w_i \Big(D_i(Z_i^L)' [\Omega_C(\beta_C)]^{-1} \sum_{j=1}^{n} U_j^C(\beta_C) + \sum_{j=1}^{n} J_{ij}^C(Z_i^L) \Big) + o_p(\frac{1}{\sqrt{n}})| \\ \leq& |\frac{1}{\sqrt{n}} \Big(\sum_{i=1}^{n} \alpha_i(x) w_i D_i(Z_i^L) \Big)` [\Omega_C(\beta_C)]^{-1} \Big(\frac{1}{\sqrt{n}} \sum_{j=1}^{n} U_j^C(\beta_C) \Big)| \\ &+ |\frac{1}{n} \sum_{i,j=1}^{n} \alpha_i(x) w_i J_{ij}^C(Z_i^L)| + o_p(\frac{1}{\sqrt{n}}) \end{aligned} \tag{S4}$$

We first analyze the first term of Eq.(S4). Notice that

$$D_i(Z_i^L) = \int_0^{Z_i^L} (X_i - \bar{x}(u;\beta_C)) \lambda_i^C(u) du = \int_0^L (X_i - \bar{x}(u;\beta_C)) \lambda_i^C(u) R_i(u) du.$$

Then

$$\sum_{i=1}^{n} \alpha_i(x) w_i D_i(Z_i^L) = \int_0^L \Big(\sum_{i=1}^{n} \alpha_i(x) w_i (X_i - \bar{x}(u;\beta_C)) \lambda_i^C(u) R_i(u) \Big) du$$

For any u, and for any fixed $\delta > 0$, the following uniformly holds for all x and u.

$$
\begin{aligned}
&E[\big|w_i(X_i - \bar{x}(u;\beta_C))\lambda_i^C(u)R_i(u) - E[w_i(X_i - \bar{x}(u;\beta_C))\lambda_i^C(u)R_i(u)|X_i = x]\big|^{2+\delta}|X_i = x] \\
=&\big(\lambda_0^C(u)\exp(\beta_C' X_i)(|X_i - \bar{x}(u;\beta_C)|)\epsilon_L\big)^{2+\delta} E[\big|\frac{w_i}{\epsilon_L}R_i(u) - E[\frac{w_i}{\epsilon_L}R_i(u)|X_i = x]\big|^{2+\delta}|X_i = x] \\
\leq&\big(\lambda_0^C(u)\exp(\beta_C' X_i)(|X_i - \bar{x}(u;\beta_C)|)\epsilon_L\big)^{2+\delta} E[\big|\frac{w_i}{\epsilon_L}R_i(u) - E[\frac{w_i}{\epsilon_L}R_i(u)|X_i = x]\big|^{2}|X_i = x] \\
\leq&\big(\lambda_0^C(u)\exp(\beta_C' X_i)(|X_i - \bar{x}(u;\beta_C)|)\epsilon_L\big)^{2+\delta}
\end{aligned}
$$

where the third line is because $\frac{w_i}{\epsilon_L}R_i(u)|X_i = x$ is a Bernoulli random variable, the fourth line is because variance of Bernoulli random variable bounded by 1. Under Assumption.4, 5, 6, 8, the k-th entry of above quantity is bound by $\big(\lambda_0^C \exp(2||\beta_C||_1 M_X) M_X \epsilon_L\big)^{2+\delta}$. Then by theorem 8 of Wager and Athey (2015),

$$
\sum_{i=1}^{n} \alpha_i(x) w_i D_i(Z_i^L) = E[w_i D_i(Z_i^L)|X_i = x] + o_p(1) = E[D_i(T_i^L)|X_i = x] + o_p(1)
$$

because it can be regarded as the random forest with responses $w_i D_i(Z_i^L)$. Someone may be confused about this result because we do not construct $\alpha_i(x)$ by $w_i D_i(Z_i^L)$, but instead by estimating equations. The trick of this result is that it does not rely on a lot about informative splitting. The prediction power is more from random(not very informative) but deep trees than informative splitting. Not very informative but deep trees are good enough reduce bias(see Lemma 1 in Wager and Athey (2015)), and variability quantification relies on a lot on random trees(see Lemma 3.2, 3.3 in Wager and Athey (2015)). Once the underlying truth satisfies smooth conditions and the tree construction follows conditions 'random-split', 'α-regular', and 'honesty', then the predictors can achieve the asymptotic normality. And $\left(\frac{1}{\sqrt{n}}\sum_{j=1}^{n} U_j^C(\beta_C)\right) = O_p(1)$ by CLT. For the second term of Eq.(S4), we first notice

$$
J_{ij}^C(Z_i^L) = \int_0^L \exp(\beta_C' X_i) R_i(u)[r^{(0)}(u,\beta_C)]^{-1} dM_j^C(u)
$$

Then

$$
\begin{aligned}
&\frac{1}{n}\sum_{i,j=1}^{n} \alpha_i(x) w_i J_{ij}^C(Z_i^L) \\
=&\frac{1}{n}\sum_{i,j=1}^{n} \alpha_i(x) w_i \left(\int_0^L \exp(\beta_C' X_i) R_i(u)[r^{(0)}(u,\beta_C)]^{-1} dM_j^C(u)\right) \qquad \text{(S5)} \\
=&\frac{1}{n}\sum_{j=1}^{n} \int_0^L \left(\sum_{i=1}^{n} \alpha_i(x) w_i \exp(\beta_C' X_i) R_i(u)\right)[r^{(0)}(u,\beta_C)]^{-1} dM_j^C(u)
\end{aligned}
$$

Since for any u, and for any fixed $\delta > 0$,

$$\begin{aligned}
&E[\big|w_i \exp(\beta_C' X_i)R_i(u) - E[w_i \exp(\beta_C' X_i)R_i(u)|X_i = x]\big|^{2+\delta}|X_i = x] \\
=&\big(\exp(\beta_C' X_i)\epsilon_L\big)^{2+\delta} E[\big|\frac{w_i}{\epsilon_L}R_i(u) - E[\frac{w_i}{\epsilon_L}R_i(u)|X_i = x]\big|^{2+\delta}|X_i = x] \\
\leq&\big(\exp(\beta_C' X_i)\epsilon_L\big)^{2+\delta} E[\big|\frac{w_i}{\epsilon_L}R_i(u) - E[\frac{w_i}{\epsilon_L}R_i(u)|X_i = x]\big|^{2}|X_i = x] \\
\leq&\big(\exp(\beta_C' X_i)\epsilon_L\big)^{2+\delta} \\
\leq&\big(\exp(||\beta_C||_1 M_X)\epsilon_L\big)^{2+\delta}
\end{aligned}$$

which uniformly holds for all x and u. $\sum_{i=1}^n \alpha_i(x) w_i \exp(\beta_C' X_i)R_i(u) = E[w_i \exp(\beta_C' X_i)R_i(u)|X_i = x] + o_p(1)$ by theorem 8 of Wager and Athey (2015). Then by CLT, we have

$$\begin{aligned}
&\frac{1}{n}\sum_{i,j=1}^{n} \alpha_i(x) w_i J_{ij}^C(Z_i^L) \\
=&\frac{1}{n}\sum_{j=1}^{n}\int_0^L \Big(E[w_i \exp(\beta_C' X_i)R_i(u)|X_i = x] + o_p(1)\Big)[r^{(0)}(u,\beta_C)]^{-1} dM_j^C(u) \\
=&O_p(\frac{1}{\sqrt{n}})
\end{aligned}$$

So in total, we have $|b_n - b_n'| = O_p(\frac{1}{\sqrt{n}})$. Similarly, we can show $|a_n - a_n'| = O_p(\frac{1}{\sqrt{n}})$.

To prove (S2), by definition, $b_n = \sum_{i=1}^n \alpha_i(x) w_i = E[w_i|X_i = x] + o_p(1) = 1 + o_p(1)$, because $E[|w_i - E[w_i|X_i = x]|^{2+\delta} X_i = x] \leq \epsilon_L^{2+\delta}$ for any fixed $\delta > 0$. By (S1), $b_n' = 1 + o_p(1)$ and $|a_n - a_n'| = O_p(\frac{1}{\sqrt{n}})$. So we have $\frac{|a_n - a_n'|}{b_n'} = O_p(\frac{1}{\sqrt{n}})$. $\frac{|b_n - b_n'|}{b_n'} = O_p(\frac{1}{\sqrt{n}})$ follows similarly.

To prove (S3), from Lemma.2, $\tilde{\mu}^L(x) = \frac{a_n}{b_n} = \mu^L(x) + O_p([\text{ploy}(\log(\frac{n}{s}))]^{-\frac{1}{2}}\sqrt{\frac{s}{n}})$. Then

$$\begin{aligned}
\tilde{\mu}^L(x) - \hat{\mu}^L(x) &= \frac{a_n b_n' - a_n b_n + a_n b_n - a_n' b_n}{b_n b_n'} \\
&= \frac{a_n}{b_n}\frac{b_n - b_n'}{b_n'} + \frac{a_n - a_n'}{b_n'} \\
&= \Big(\mu^L(x) + O_p([\text{ploy}(\log(\frac{n}{s}))]^{-\frac{1}{2}}\sqrt{\frac{s}{n}}\Big) O_p(\frac{1}{\sqrt{n}}) + O_p(\frac{1}{\sqrt{n}}) \\
&= O_p(\frac{1}{\sqrt{n}})
\end{aligned}$$

Combine (S1), (S2), (S3), and $s = n^\beta$, we have

$$\frac{\tilde{\mu}^L(x) - \hat{\mu}^L(x)}{\sigma_n(x)} = O_p(\frac{1}{\sqrt{n}}) O_p([\text{ploy}(\log(\frac{n}{s}))]^{\frac{1}{2}}\sqrt{\frac{n}{s}} = o_p(1)$$

because $\text{poly}\Bigg(\log(\frac{n}{s})\Bigg) = \text{poly}(\log n)$.

The result below verifies that the optimal half-sampling estimator $\sigma_n^2(x)$ with plug-in values for $\hat{\mu}^L(x)$ consistently estimates the sampling variance of $\Psi(\mu^L(x))$. We

LEMMA 5. *Under assumption of Lemma.2,*

$$\frac{|\hat{\sigma}_n^2(x) - \bar{\sigma}_n^2(x)|}{\bar{\sigma}_n^2(x)} \rightarrow_p 0$$

PROOF OF LEMMA.5. Following the argument of Athey et al. (2018), we consider the expectation when number of trees $B \rightarrow \infty$,

$$E[\hat{\sigma}_n^2(x)] = E[(\Psi_{\mathcal{H}}(\hat{\mu}^L(x)) - \Psi(\hat{\mu}^L(x)))^2]$$

for $\mathcal{H} = 1, ..., n/2$.

$$\begin{aligned}
&\Psi_{\mathcal{H}}(\hat{\mu}^L(x)) - \Psi(\hat{\mu}^L(x)) \\
=&\Bigg(\tilde{\Psi}_{\mathcal{H}}(\tilde{\mu}^L(x)) - \tilde{\Psi}(\tilde{\mu}^L(x))\Bigg) \\
&-\Bigg(\Psi(\hat{\mu}^L(x)) - \tilde{\Psi}(\hat{\mu}^L(x)) + \tilde{\Psi}(\hat{\mu}^L(x)) - \tilde{\Psi}(\tilde{\mu}^L(x))\Bigg) \\
&+\Bigg(\Psi_{\mathcal{H}}(\hat{\mu}^L(x)) - \tilde{\Psi}_{\mathcal{H}}(\hat{\mu}^L(x)) + \tilde{\Psi}_{\mathcal{H}}(\hat{\mu}^L(x)) - \tilde{\Psi}_{\mathcal{H}}(\tilde{\mu}^L(x))\Bigg) \\
=&A + B + C
\end{aligned}$$

Term A is estimating equation contains true IPCW, and the plug-in parameter estimators is also evaluated under true IPCW. For term B, using quantities defined in Lemma.4,

$$\begin{aligned}
|B| =&|\Psi(\hat{\mu}^L(x)) - \tilde{\Psi}(\hat{\mu}^L(x)) + \tilde{\Psi}(\hat{\mu}^L(x)) - \tilde{\Psi}(\tilde{\mu}^L(x))| \\
=&\Bigg|\big(\sum_{i=1}^{n} \alpha_i(x)(\hat{w}_i - w_i)(Z_i^L - \hat{\mu}^L(x))\big) + (\hat{\mu}^L(x) - \tilde{\mu}^L(x))\big(\sum_{i=1}^{n} \alpha_i(x) w_i\big)\Bigg| \\
=&|(a_n - a_n') - \hat{\mu}^L(x)(b_n - b_n') + (\hat{\mu}^L(x) - \tilde{\mu}^L(x))(1 + o_p(1))| \\
\leq&|O_p(\frac{1}{\sqrt{n}}) + (\mu^L(x) + O_p(\frac{1}{\sqrt{n}}))O_p(\frac{1}{\sqrt{n}}) + O_p(\frac{1}{\sqrt{n}})(1 + o_p(1))| \\
=&O_p(\frac{1}{\sqrt{n}})
\end{aligned}$$

Similarly, C also has order $O_p(\frac{1}{\sqrt{n}})$. By Theorem.5, 6 in Athey et al. (2018), we have $Var[A] = (1 + o(1))\bar{\sigma}_n^2 = \text{polylog}(n/s)^{-1}(s/n)$. By Cauchy-Schwarz inequality, we conclude that $|E[\hat{\sigma}_n^2(x)] - \bar{\sigma}_n^2|/\bar{\sigma}_n^2 = o_p(1)$

PROOF OF THEOREM.1. The key idea is to write the quantity of interest into the following form

$$\frac{\hat{\mu}^L(x) - \mu^L(x)}{\hat{\sigma}_n(x)} = \frac{\bar{\sigma}_n(x)}{\hat{\sigma}_n(x)}\Bigg(\frac{\hat{\mu}^L(x) - \tilde{\mu}^L(x)}{\bar{\sigma}_n(x)} + \frac{\tilde{\mu}^L(x) - \mu^L(x)}{\bar{\sigma}_n(x)}\Bigg). \tag{S6}$$

We show that first term is $1 + o_p(1)$ in Lemma 5 with continuous-mapping theorem, the second term is $o_p(1)$ in Lemma 3 and Lemma 4, and the third term is asymptotic normal in Lemma 1 and Lemma.2. Theorem 1 is then proved by Slutsky's theorem.

2 ADDITIONAL SIMULATION RESULTS

Figure S1, Figure S2 and Figure S3 show results of comparison of performance of multiple methods under identity link, logexp link and exp lin, respectively. The first column is $p = 5, 10, 20$ for Model 1 and the second column is $p = 5, 10, 20$ for Model 2..

3 ADDITIONAL RESULTS ON TCGA DATA ANALYSIS

Performance of the proposed random forest estimator compared with other methods for $L = 1, 3, 5, 7, 9, 11$. The left side is MAE across of 10-fold cross-validation. The right side is RMSE across of 10-fold cross-validation. We shuffle the data 20 times and take the mean of MAE and RMSE.

REFERENCES

Athey, S., Tibshirani, J., and Wager, S. (2018). *Generalized Random Forests*. Tech. Rep. 1

Tian, L., Zhao, L., and Wei, L. J. (2014). Predicting the restricted mean event time with the subject's baseline covariates in survival analysis. *Biostatistics* 15, 222–233. doi:10.1093/biostatistics/kxt050

Wager, S. and Athey, S. (2015). Estimation and Inference of Heterogeneous Treatment Effects using Random Forests doi:10.1073/pnas.1510489113

Wang, X. and Schaubel, D. E. (2018). Modeling restricted mean survival time under general censoring mechanisms. *Lifetime Data Analysis* 24, 176–199. doi:10.1007/s10985-017-9391-6

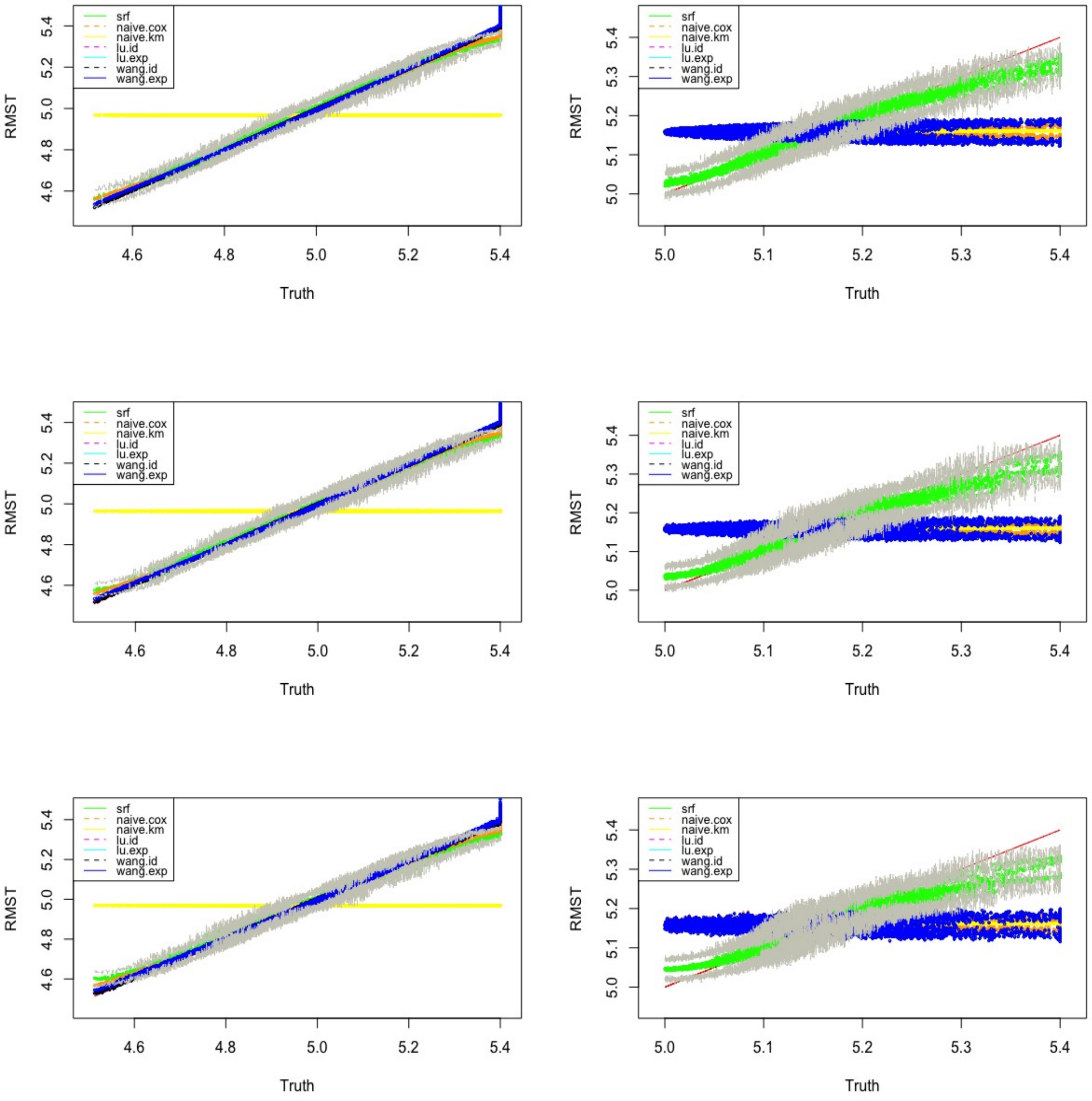

Figure S1. Estimated versus the true RMST for Model 1 (left panel) and Model 2 (right panel) with identity link function and the number of covariates $p = 5, 10, 20$ (top to bottom). SRF: proposed random forest-bases estimator, and upper and lower bounds of the point-wise confidence intervals of the proposed random forest estimator are connected in the grey lines; Naive.km: estimate based on Kaplan-Meier estimator without adjusting for the covariates; Naive.Cox: Cox regression based estimator; Lu.id: method of Tian et al. (2014) with identity link; Lu.exp: method of Tian et al. (2014) with exponential link; Wang.id: method of Wang and Schaubel (2018) with identity link; Wang:exp: method of Wang and Schaubel (2018) with exponential link.

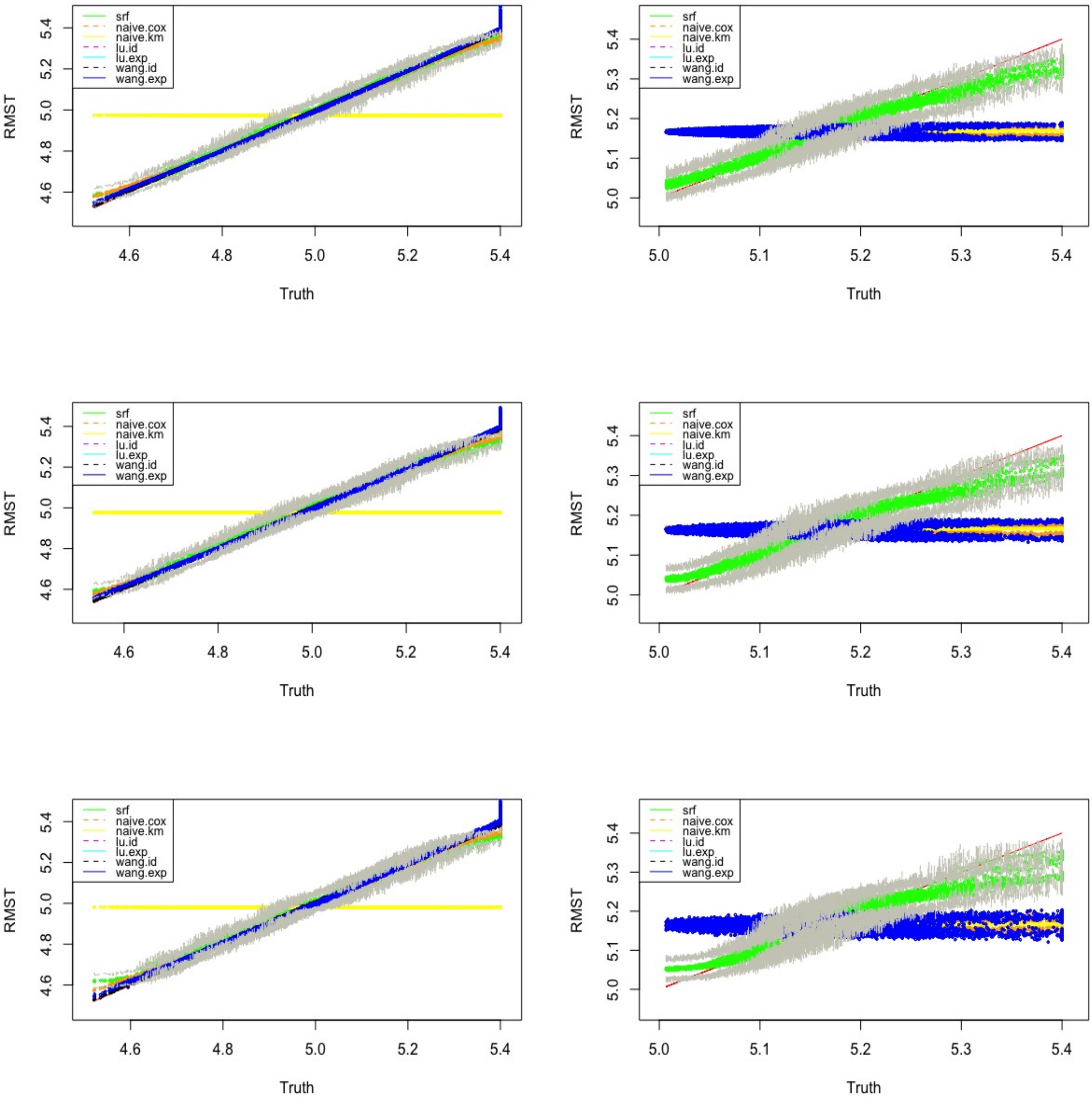

Figure S2. Estimated versus the true RMST for Model 1 (left panel) and Model 2 (right panel) with log-exponential link function and the number of covariates $p = 5, 10, 20$ (top to bottom). SRF: proposed random forest-bases estimator, and upper and lower bounds of the point-wise confidence intervals of the proposed random forest estimator are connected in the grey lines; Naive.km: estimate based on Kaplan-Meier estimator without adjusting for the covariates; Naive.Cox: Cox regression based estimator; Lu.id: method of Tian et al. (2014) with identity link; Lu.exp: method of Tian et al. (2014) with exponential link; Wang.id: method of Wang and Schaubel (2018) with identity link; Wang:exp: method of Wang and Schaubel (2018) with exponential link.

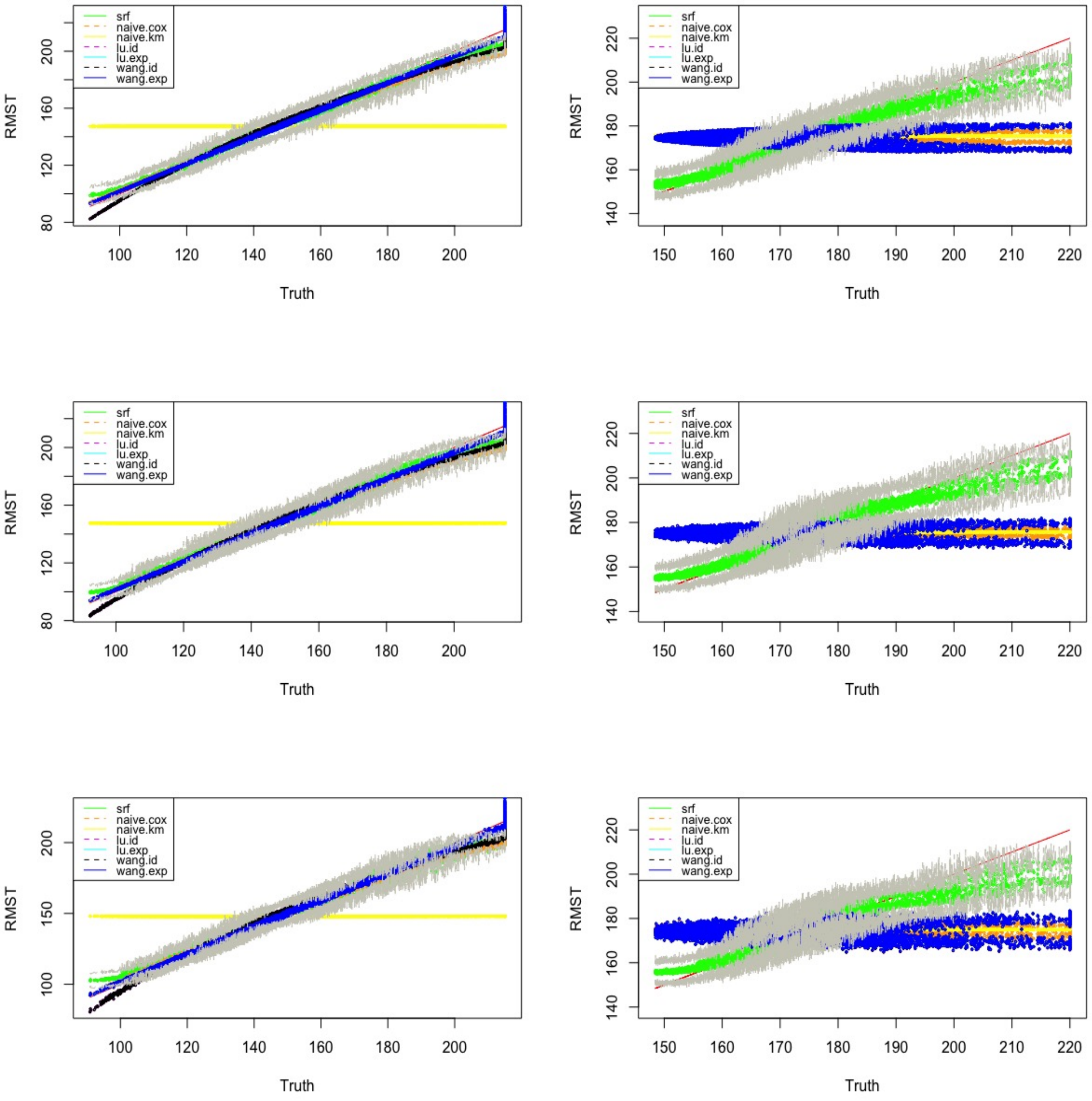

Figure S3. Estimated versus the true RMST for Model 1 (left panel) and Model 2 (right panel) with exponential link function and the number of covariates $p = 5, 10, 20$ (top to bottom). SRF: proposed random forest-bases estimator, and upper and lower bounds of the point-wise confidence intervals of the proposed random forest estimator are connected in the grey lines; Naive.km: estimate based on Kaplan-Meier estimator without adjusting for the covariates; Naive.Cox: Cox regression based estimator; Lu.id: method of Tian et al. (2014) with identity link; Lu.exp: method of Tian et al. (2014) with exponential link; Wang.id: method of Wang and Schaubel (2018) with identity link; Wang:exp: method of Wang and Schaubel (2018) with exponential link.

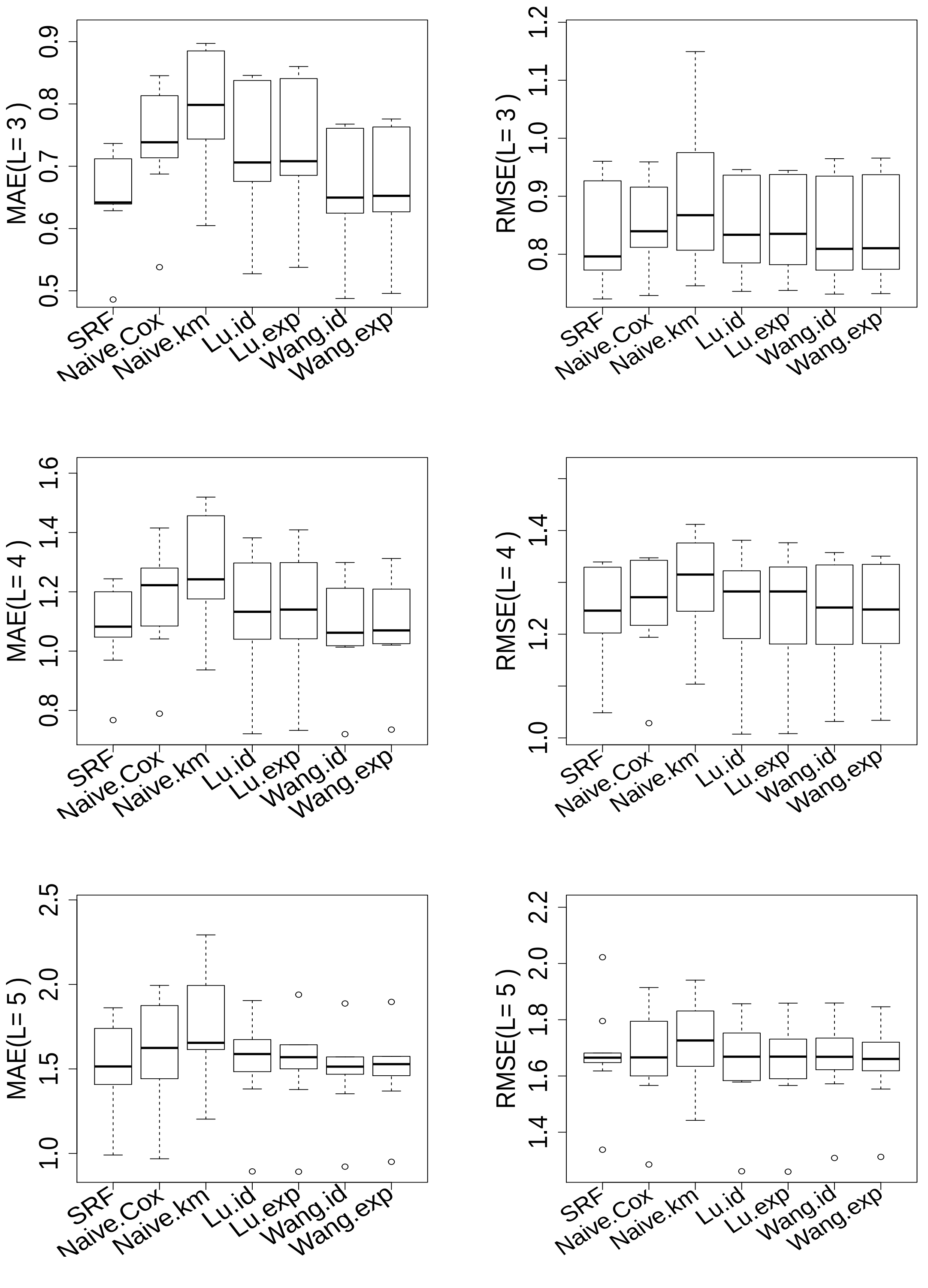

Figure S4. Performance of the proposed random forest estimator compared with other methods for $L = 3, 4, 5$. The left penal is the MAE across of 10-fold cross-validation. The right panel is the RMSE across of 10-fold cross-validation. SRF: proposed random forest estimator; Naive.km: estimate based on Kaplan-Meier estimator without adjusting for the covariates; Naive.Cox: Cox regression based estimator; Lu.id: method of Tian et al. (2014) with identity link; Lu.exp: method of Tian et al. (2014) with exponential link; Wang.id: method of (Wang and Schaubel, 2018) with identity link; Wang:exp: method of Wang and Schaubel (2018) with exponential link.

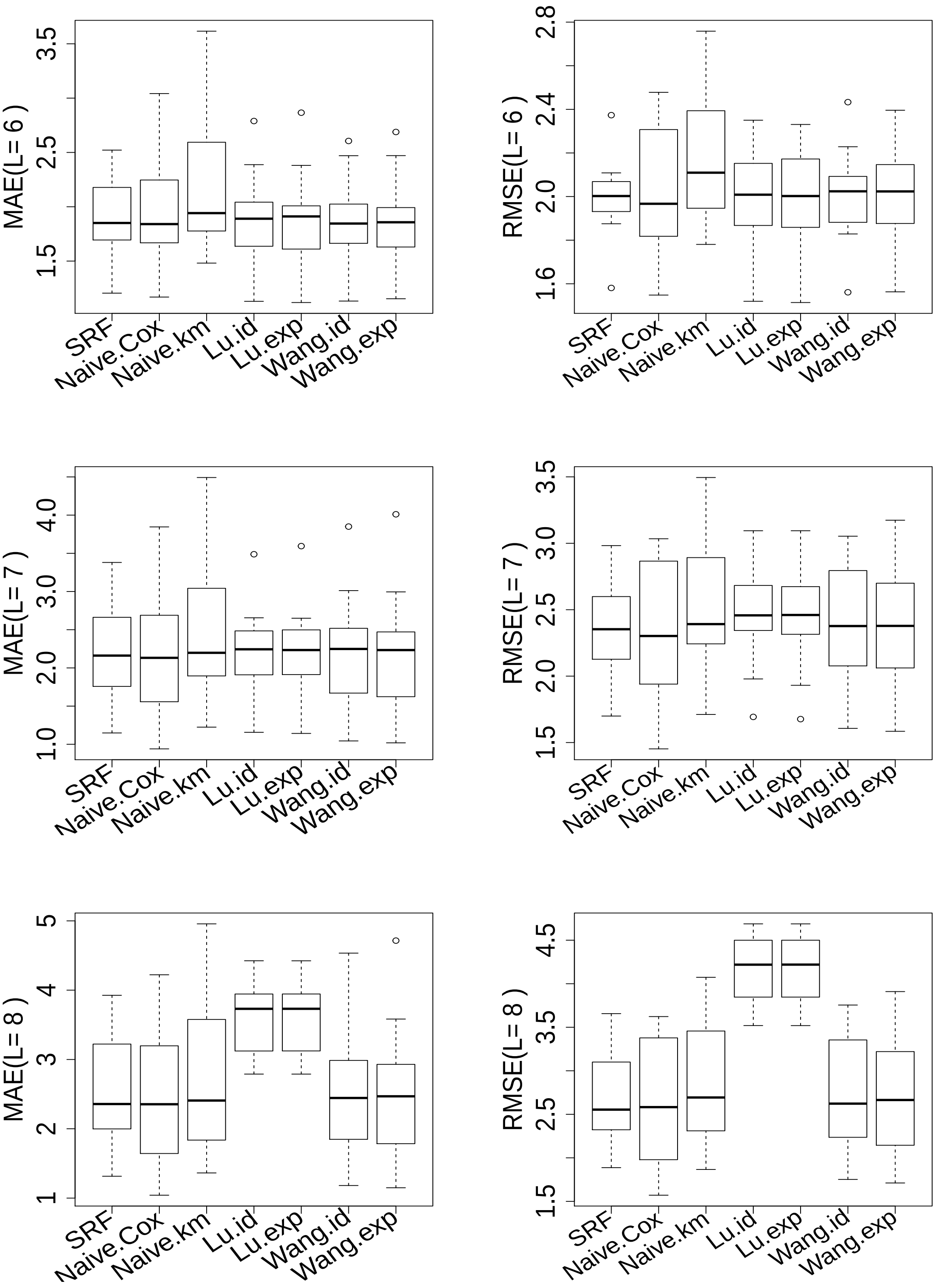

Figure S5. Performance of the proposed random forest estimator compared with other methods for $L = 6, 7, 8$. The left penal is the MAE across of 10-fold cross-validation. The right panel is the RMSE across of 10-fold cross-validation. SRF: proposed random forest estimator; Naive.km: estimate based on Kaplan-Meier estimator without adjusting for the covariates; Naive.Cox: Cox regression based estimator; Lu.id: method of Tian et al. (2014) with identity link; Lu.exp: method of Tian et al. (2014) with exponential link; Wang.id: method of (Wang and Schaubel, 2018) with identity link; Wang:exp: method of Wang and Schaubel (2018) with exponential link.
